# Functional Analysis of the Chaperone-Usher Fimbrial Gene Clusters of *Salmonella enterica* serovar Typhi

**DOI:** 10.3389/fcimb.2018.00026

**Published:** 2018-02-08

**Authors:** Karine Dufresne, Julie Saulnier-Bellemare, France Daigle

**Affiliations:** Department of Microbiology, Infectiology and Immunology, Université de Montréal, Montreal, QC, Canada

**Keywords:** chaperone-usher, fimbriae, *S. Typhi*, infection, biofilm, pathogenesis

## Abstract

The human-specific pathogen *Salmonella enterica* serovar Typhi causes typhoid, a major public health issue in developing countries. Several aspects of its pathogenesis are still poorly understood. *S*. Typhi possesses 14 fimbrial gene clusters including 12 chaperone-usher fimbriae (*stg, sth, bcf*, *fim, saf*, *sef*, *sta, stb, stc, std, ste*, and *tcf*). These fimbriae are weakly expressed in laboratory conditions and only a few are actually characterized. In this study, expression of all *S*. Typhi chaperone-usher fimbriae and their potential roles in pathogenesis such as interaction with host cells, motility, or biofilm formation were assessed. All *S*. Typhi fimbriae were better expressed in minimal broth. Each system was overexpressed and only the fimbrial gene clusters without pseudogenes demonstrated a putative major subunits of about 17 kDa on SDS-PAGE. Six of these (Fim, Saf, Sta, Stb, Std, and Tcf) also show extracellular structure by electron microscopy. The impact of fimbrial deletion in a wild-type strain or addition of each individual fimbrial system to an *S*. Typhi afimbrial strain were tested for interactions with host cells, biofilm formation and motility. Several fimbriae modified bacterial interactions with human cells (THP-1 and INT-407) and biofilm formation. However, only Fim fimbriae had a deleterious effect on motility when overexpressed. Overall, chaperone-usher fimbriae seem to be an important part of the balance between the different steps (motility, adhesion, host invasion and persistence) of *S*. Typhi pathogenesis.

## Introduction

*Salmonella enterica* serovar Typhi is a human-specific pathogen responsible for a systemic disease called typhoid fever. It causes ~22 million infections and 200,000 deaths annually worldwide (WHO, 2012; Qamar et al., 2015). Over the years, the number of cases has increased, but the use of antibiotics has controlled the eventual burden. However, the increased emergence of multidrug resistant *S*. Typhi strains can complicate treatment and can lead to a higher death rate (Rowe et al., 1997; Thong et al., 2000; Pokharel et al., 2006; WHO, 2012). A better understanding of *S*. Typhi pathogenesis is required to better control and treat typhoid (Obaro et al., 2017).

*Salmonella* species enter their host by the intestinal tract and cross the intestinal barrier (Clark et al., 1994). *S*. Typhi invades the human host by a variety of virulence factors such as two type III secretion systems (T3SS) encoded by *Salmonella* pathogenicity islands (SPI)−1 and−2, the presence of 10 SPIs in the genome, the human-restricted typhoid toxin and the flagella (Galan and Zhou, 2000; Galán, 2001; Waterman and Holden, 2003; Chang et al., 2016; Horstmann et al., 2017). It also evades the host innate immune response by the production of an extracellular capsule, the Vi antigen, encoded on SPI-7 (Wilson et al., 2008; Winter et al., 2008; Wangdi et al., 2014). Most of the data available about *S*. Typhi pathogenesis and virulence factors is based on the systemic infection of mice with *S*. Typhimurium. Due to this lack of direct information, crucial questions still remain concerning host-specificity and pathogenicity mechanisms of *S*. Typhi.

**Figure 1:**
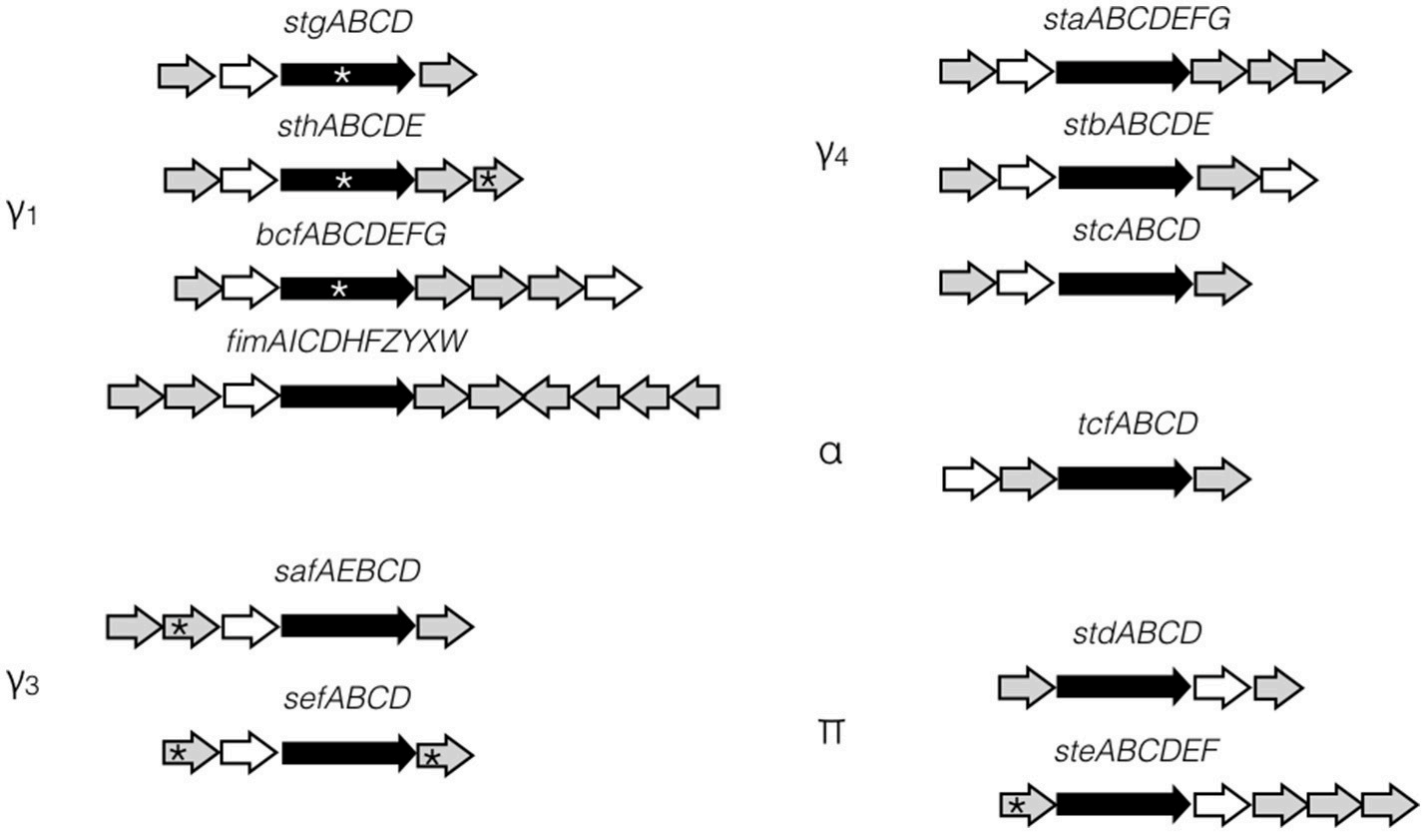
*S*. Typhi CU fimbrial operons organization. *S*. Typhi possesses 12 putative fimbrial gene clusters divided in 5 clades (γ_1_, γ_3_, γ_4_, α, and π) depending of the usher homologies. Stg, Sth, Bcf, and Fim are γ_1_ fimbriae, while Saf and Sef are in clade γ_3_ and Sta, Stb, and Stc are in clade γ_4_. Tcf is the only representative of α clade. Std and Ste are π fimbriae. Ushers are represented by black arrow and chaperones by white arrow. No distinction is made for major/minor subunits, adhesins, or other proteins (gray arrows). Pseudogenes are marked by asterisk.

Fimbriae are proteinaceous extracellular structures mainly involved in adhesion, a crucial initial step for colonization and entry into host cells. Fimbriae have also been shown to contribute to interactions with macrophages, intestinal persistence, biofilm formation and bacterial aggregation in other *Salmonella* serovars (Edwards et al., 2000; Zhang et al., 2000; Boddicker et al., 2002; Tsui et al., 2003; Weening et al., 2005; Ledeboer et al., 2006). Fimbriae are grouped into three classes according to their mode of assembly. The curli fimbriae are assembled by a process called nucleation-precipitation where the major subunits are precipitated together by the presence of the nucleator in the extracellular medium. The type IV fimbriae are assembled at the inner-membrane platform and extended through the periplasm and outer membrane to the extracellular environment. This fimbria can be assembled or disassembled using ATP. Lastly, the chaperone-usher (CU) fimbriae use a periplasmic chaperone and an outer-membrane usher to assemble the major subunits into the final external filamentous structures. This class of fimbriae is the most diverse and *S*. Typhi fimbriae are phylogenetically sub-classified into five clades based on the usher: γ_1_, γ_3_, γ_4_, π, and α (Figure 1; Townsend et al., 2001; Nuccio and Bäumler, 2007).

The *S*. Typhi genome possesses a unique repertoire of 14 putative fimbrial clusters identified by whole-genome sequencing (Humphries et al., 2003) including 12 CU fimbriae (Townsend et al., 2001). However, only 3 CU fimbriae (Stg, Sta, and Tcf) were previously studied (Forest et al., 2007; Bishop et al., 2008; Berrocal et al., 2015; Leclerc et al., 2016; Gonzales et al., 2017), mainly due to the weak expression of most of these fimbriae under laboratory conditions (Low et al., 2006; De Masi et al., 2017). Among the CU fimbriae, 5 (*stg, sef*, *sta, ste*, and *tcf*) are present in *S*. Typhi but absent in the well-studied broad-range pathogen *S*. Typhimurium and 5 of the fimbrial gene clusters in *S*. Typhi (*stg, sth, bcf*, *sef*, and *ste*) have at least one pseudogene. Approximately 5% of the *S*. Typhi genome contains pseudogenes, which were often associated with its host-specificity and may restrict *S*. Typhi only to the human host (Baker and Dougan, 2007). In fimbrial putative gene clusters, pseudogenes are present in the usher genes (*stgC, sthC*, and *bcfC*), but also in subunits or adhesin genes (*sthE, sefA, sefD*, and *steA*). However, *S*. Typhi mutants with a deletion of *stg* demonstrated decreased infection of cell lines, suggesting a potential function for this fimbrial cluster despite the presence of a pseudogene in the usher gene (Forest et al., 2007; Berrocal et al., 2015; Gonzales et al., 2017). Allelic variation, especially for the FimH adhesin of the Type I fimbria, was studied in several serovars and may also be implicated in host tropism for *Salmonella* (Kisiela et al., 2012; Yue et al., 2015; De Masi et al., 2017).

Here, we hypothesize that some of 12 CU fimbriae of *S*. Typhi are produced and involved at different steps of pathogenesis despite their poor fimbrial expression and presence of pseudogenes. The characterization of all 12 CU fimbriae of *S*. Typhi includes expression levels under the tested conditions, surface structure assembly, interactions with host cells, role in biofilm production and in motility. Overall, each of the *S*. Typhi fimbrial systems were found to contribute to different steps of the pathogenesis process and six of these produced visible fimbriae.

## Materials and methods

### Bacterial strains, plasmids, and growth conditions

The list of bacterial strains and vectors used in this study is given in Table S1. Bacteria were routinely grown overnight at 37°C on Luria-Bertani (LB) agar plates or with agitation in LB broth. When required, supplements or antibiotics were added at the following concentrations: 0.05–1 mM IPTG, 50 μg/ml diaminopimelic acid (DAP), 50 μg/ml kanamycin, 50 μg/ml ampicillin, and 34 μg/ml chloramphenicol. IPTG is used to induce expression of fimbrial cluster cloned into pMMB307c (Morales et al., 1991) and DAP is an amino acid that allows the maintain of the conjugative strain MGN-617 containing an *asd* mutation (Kaniga et al., 1991). Transformation of bacteria was performed by using the calcium/manganese based (CCMB) or electroporation methods as previously described (O'Callaghan and Charbit, 1990).

### Cloning of fimbrial promoters and β-galactosidase assays

The primers used for cloning of fimbrial promoters are listed in Table S2. The promoter region upstream of each gene cluster was predicted by the Softberry software BPROM (www.softberry.com) and amplified by PCR reaction. PCR fragments between 170 and 730 bp were cloned upstream of the promoterless *lacZ* gene in vector pRS415. The resulting vector was transformed into *S*. Typhi wild-type strain. Expression of each promoter was measured by β-galactosidase assays following growth under different culture conditions. LB was used as a classic rich laboratory medium and bacteria were inoculated in broth or on agar and incubated overnight at 37°C. M63 was used as a minimal medium and was prepared as previously described (Leclerc et al., 2013). Bacteria were inoculated in M63 broth or on M63 agar. For the induction of the T3SS encoded on SPI-1, the bacteria were grown in LB 0.3M NaCl, and incubated overnight at 37°C without agitation for low oxygenation (Lee et al., 1992; Weinstein et al., 1998). For the induction of T3SS encoded on SPI-2, the bacteria were incubated in LPM broth, pH 5.8, and incubated overnight at 37°C with agitation (Coombes et al., 2004). For each condition, β-galactosidase activity was assessed using *o*-nitrophenyl-β-D-galactopyranoside (ONPG) as described previously (Miller, 1972).

### Chromosomal deletion of fimbrial gene clusters

The primers used for mutagenesis are listed in Table S2. Mutant strains for each fimbrial gene cluster were obtained by allelic exchange mutagenesis as previously described (Forest et al., 2007) except for the deletion of *sta*, that was obtained by λ red recombination system (Datsenko and Wanner, 2000). Each fimbrial deletion was verified by PCR (data not shown). The afimbrial strain resulted from the successive deletion of each of the fimbrial clusters, including the deletion of genes encoding the curli (*csg*) and the type IV fimbriae (*pil*). At least two different mutants were tested for each construction and no major phenotypic differences (growth curves and adhesion assay) were observed. Only one mutant was then selected for further experiments.

### Cloning of the fimbrial gene clusters

The primers used for cloning of fimbrial gene clusters are listed in Table S2. Fimbrial gene clusters, with or without the promoter region, were amplified by PCR using Q5 High-Fidelity DNA polymerase (New England Biolabs). Fragments between 4.5 and 9.7 Kb were then cloned in IPTG-inducible vector pMMB207c (fimbrial gene cluster without promoter region) (Morales et al., 1991) or into the low-copy vector pWSK29 (fimbrial gene cluster with its native promoter region) (Wang and Kushner, 1991). These constructions were transformed by electroporation into the afimbrial *S*. Typhi strain.

### SDS-PAGE and transmission electron microscopy

*S*. Typhi containing the inducible vector pMMB207c with or without each fimbrial gene cluster was induced overnight at 37°C on LB plates with chloramphenicol and 50 μM IPTG. Bacteria were harvested in LB broth. For SDS-PAGE, bacteria were washed with a solution of 0.9% sodium chloride and then with a solution of 75 mM sodium chloride and 0.5 mM Tris pH 7.4. Extracellular proteins were extracted by heat treatment at 60°C during 15 min and were precipitated by addition of 10% trichloroacetic acid (Beloin et al., 2006). The concentration of proteins was normalized. The samples were loaded on SDS-PAGE 15% and stained with Coomassie R-250. The band of interest for Std and Stc (StdA and StcA) were cut from the gel, destained and digested by trypsin. Peptides were sequenced using LC-MS/MS at the Center for Advanced Proteomics Analyses (IRIC, Université de Montréal).

For electron microscopy, the 3–24 h-induced cultures were fixed with 2% glutaraldehyde for 30 min and adsorbed onto nickel formvar-carbon coated grids for 10 min and stained with 1% phosphotungstic acid. Bacteria were observed under Philips CM-100 or Hitachi H-7100 electron microscope.

### Interactions with human epithelial intestinal cells

INT-407 (Henle) cells (ATCC CCL-6) were grown in minimal essential medium supplemented (Wisent) with 10% heat-inactivated fetal bovine serum (FBS) (Wisent) and 25 mM HEPES (Wisent). The bacterial cells were grown overnight at 37°C in LB 0.3 M NaCl with low oxygenation (without agitation). This growth condition (microaerophilic and high salt medium) induce the T3SS encoded by SPI-1 (Weinstein et al., 1998; Elhadad et al., 2016; Jiang et al., 2017). The assays were then performed as previously described with MOI of 20 (Forest et al., 2007).

### Interactions with macrophages

THP-1 cells (ATCC TIB-202) were maintained in RPMI 1640 (Wisent) supplemented with 10% heat-inactivated FBS (Wisent), 1 mM sodium pyruvate (Wisent) and 1% MEM non-essential amino acids (Wisent). The assays were then performed as previously described at a MOI of 10 (Daigle et al., 2001).

### Biofilm assays

The protocol was adapted from Ganjali Dashti et al. (2016). Bacteria were grown overnight in LB broth. The cultures were then diluted 1:10 in nutrient broth containing bile, glucose, and potassium and incubated 72 h statically at 37°C. The biomass production was determined by the crystal violet assay as described by Tremblay et al. (2015). The assay was carried out at least three times for each bacterial strain.

### Motility assays

The motility assays were as previously described (Sabbagh et al., 2012). Bacteria were grown in LB broth overnight with agitation at 37°C and diluted 1/100 prior to puncture of the agar plate for mutant strains and afimbrial strains with pWSK29 vector. For afimbrial strains with inducible vector pMMB207c, the overnight cultures were diluted 1/100 in new medium and the strains were induced with 1 mM IPTG for 3 h at OD_600_ value of 0.6 and then punctured in the agar plate. Bacterial strains were accompanied by the control strain on each agar plate for comparison. Plates were incubated for 16 h at 37°C. The diameter (mm) was measured and each construction was tested at least in triplicates. The results are presented as the mean ratio of the tested strain/wild-type ± SEM of the replicas.

### Statistical analysis

Statistical analysis was performed on GraphPad Prism. Two-tailed unpaired Student's *t*-test was applied on data sets for each construction compared to their control data. *P* < 0.001 was considered extremely significant (^***^); *P* < 0.01 was considered very significant (^**^) and *P* < 0.05 was considered significant (^*^).

## Results

### *S*. Typhi CU fimbriae are better expressed in minimal media

**Figure 2:**
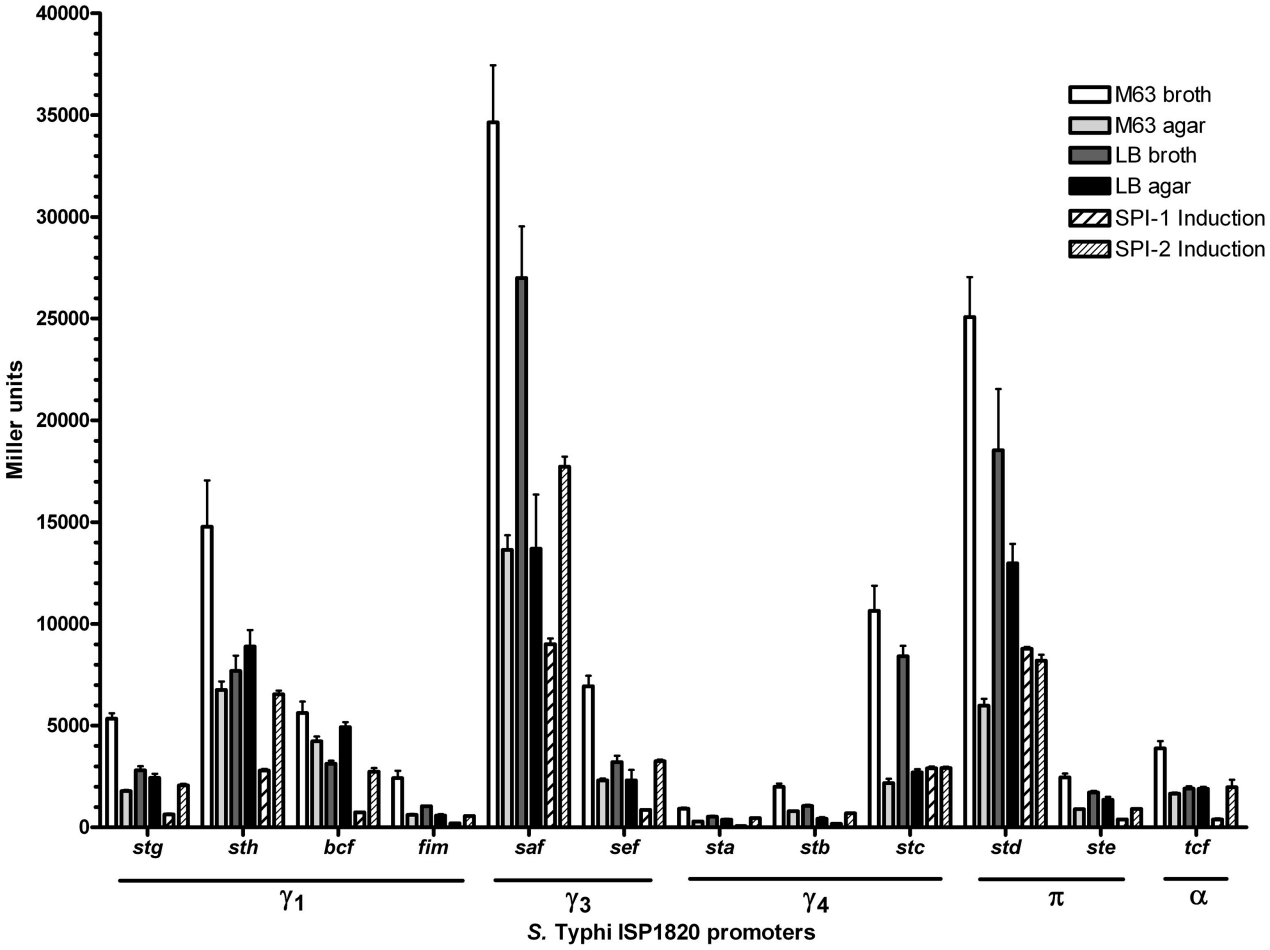
Expression of *S*. Typhi ISP1820 CU fimbrial promoters. Expression of upstream predicted promoter region was assessed by β-galactosidase assay. Six growth conditions mimicking infection were evaluated. M63 broth (white bars) and agar (light gray bars) were tested as minimal media. LB broth (dark gray bars) and agar (black bars) were presented as rich media. Media inducing SPI-1 (wide striped bars) or−2 (slim striped bars) T3SS were used. Results are expressed as the mean ± SEM of at least three distinct experiments performed in duplicates.

The first step to characterize the CU fimbriae of *S*. Typhi was to determine the best *in vitro* condition of expression of each fimbria. As most fimbriae are often poorly expressed during growth under classic laboratory conditions, each fimbrial promoter was cloned in fusion with the reporter gene *lacZ* on a multicopy vector (pRS415). The β-galactosidase activities of these transcriptional fusions were then assessed in 6 different conditions including conditions that induce the *Salmonella* T3SSs and that may mimic possible environmental cues encountered by *S*. Typhi. At first glance, the expression pattern seems similar regardless of the tested condition with the highest expression for the *saf* promoter followed by *std, sth*, and *stc*, whereas *sta, stb*, and *fim* had the lowest expression (Figure 2). All fimbrial promoters showed their highest expression in minimal medium and the lowest when SPI-1 was induced, except for *stc* and *std*. Altogether, promoters reacted to different growth conditions and demonstrated variation in their expression.

### Production and visualization of CU fimbriae in a S. Typhi afimbrial mutant

**Figure 3:**
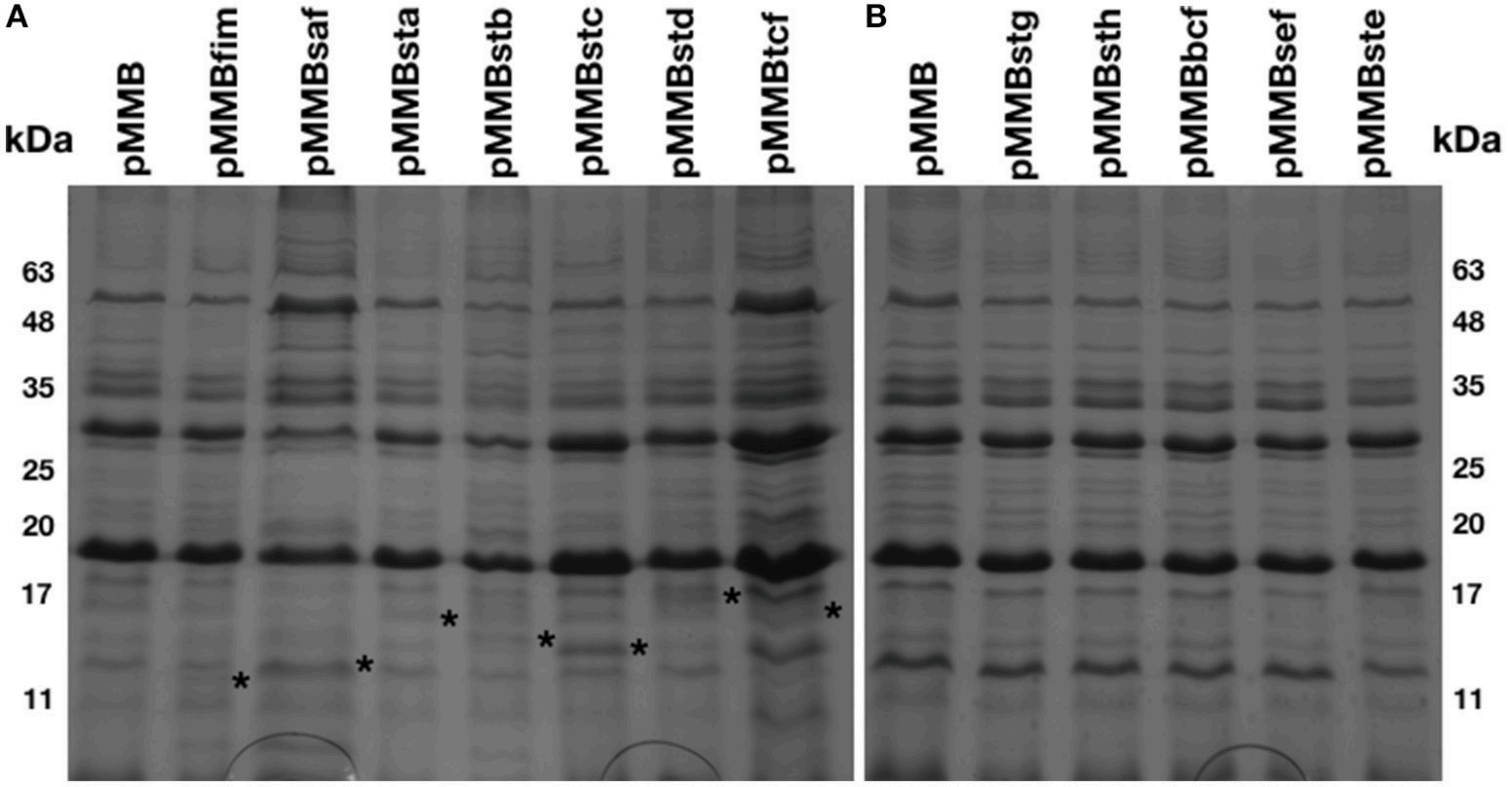
Fimbrial major subunits in extracellular structures extract. Bacteria were harvested from a LB agar plate supplemented with 50 μM IPTG and treated to extract the extracellular structures. **(A)** The control (pMMB) and 7 fimbrial gene clusters without pseudogene were migrated together on polyacrylamide gel and stained with Coomassie blue as well for **(B)** the control and the 5 fimbrial gene clusters with pseudogene. Asterisks are placed at the right of the proteinaceous bands considered as fimbrial subunits.

**Figure 4:**
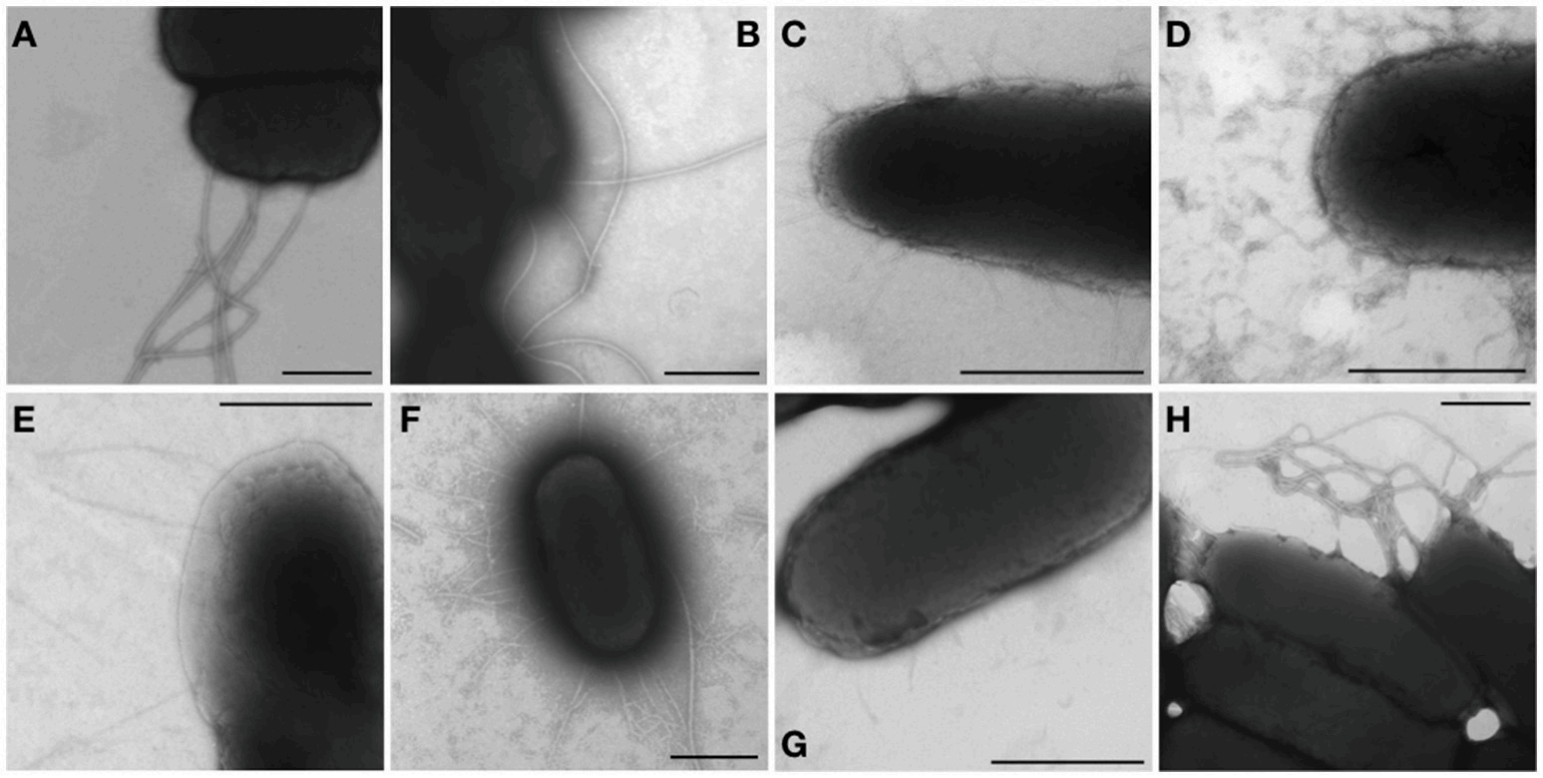
Visible fimbriae in transmission electron microscopy. Bacteria were harvested from a 24 h-induction in LB broth supplemented with 1 mM IPTG or on LB agar supplemented with 50 μM IPTG and then fixated with 2% glutaraldehyde for 30 min. The formvar-carbon grids were stained with 1% phosphotungtic acid. Native operon was cloned under lactose-inducible promoter on pMMB207c vector and transformed into afimbrial ISP1820. **(A,B)** The control (pMMB207c in wild-type and afimbrial strains respectively) presents only flagella. **(C–I)** Fimbriae of different gene clusters are represented: **(C)** Fim fimbriae, **(D)** Saf fimbriae, **(E)** Sta fimbriae, **(F)** Stb fimbriae, **(G)** Std fimbriae, and **(H)** Tcf fimbriae. Black bars = 500 nm.

An afimbrial strain of *S*. Typhi was constructed in order to eliminate functional redundancy and assess the role of each individual fimbria. Markerless and non-polar deletions of each of the 14 fimbrial gene clusters of *S*. Typhi (CU, type IVb, and curli fimbriae) were obtained by allelic exchange mutagenesis and result in an afimbrial strain harboring a total deletion of 91.5 Kb from its genome. This strain had a similar growth curve compared to the wild-type strain (data not shown). Each complete fimbrial system was overexpressed by cloning on an IPTG-inducible vector and production of fimbrial proteins was induced in the afimbrial strain. The presence of the fimbrial subunits was verified on Coomassie blue stained gel (Figure 3). The production of the major subunits of Fim, Saf, Sta, Stb, Stc, Std, and Tcf fimbrial proteins was confirmed by visualization of a band between 10 and 17 kDa that is consistent with predicted molecular mass of the mature secreted proteins. Mass spectrometry analyses identified StdA and StcA in the respective extracted bands. For Std, 53 specific peptides with 100% probability corresponding to StdA were identified, covering 167/193 amino acids, representing 87% of the predicted protein. For Stc, 43 specific peptides with 100% probability corresponding to StcA were identified, covering 131/176 amino acids, representing 74% of the predicted protein. When Fim fimbriae were induced, cells demonstrated a growth defect and lysis even at 10 μM IPTG (data not shown). No specific bands were visualized for any of the fimbrial gene clusters containing pseudogenes. Each fimbrial systems was induced for electronic microscopy analysis and the presence of Fim, Saf, Sta, Stb, Std, and Tcf was confirmed as functionally assembled fimbriae were visualized on the bacterial surface (Figure 4). Fim fimbriae are straight and short. They cover most of the bacterial cell surface. Saf fimbriae are thin and aggregated together. Sta and Stb fimbriae are straight and long, but seem shorter than flagella. Std are short and thin fimbriae. Tcf fimbriae are cable-like fimbriae and intertwine together.

### Adhesion and invasion of epithelial cells

**Figure 5:**
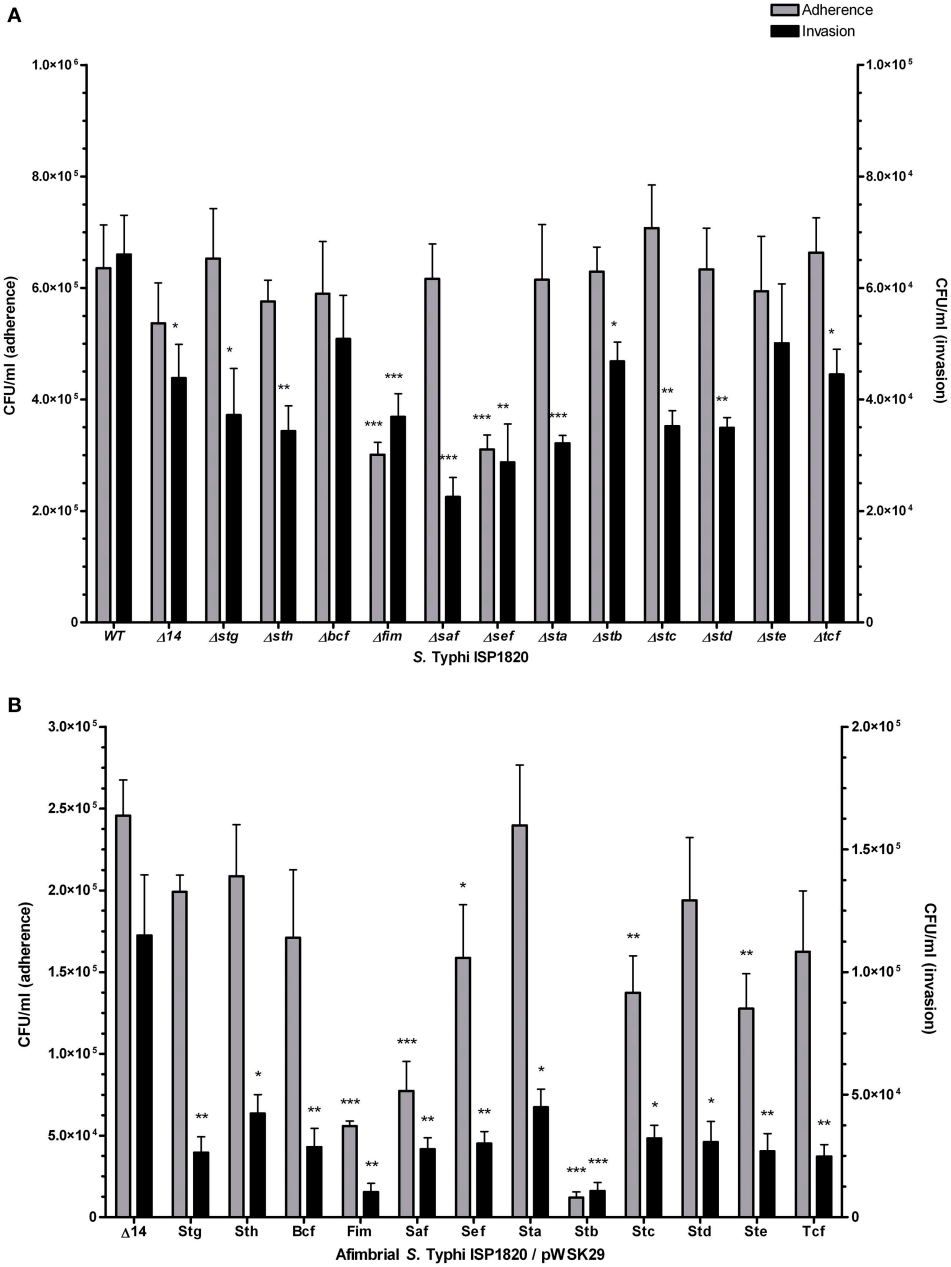
Fimbrial interactions with INT-407 intestinal epithelial cells. Adherence (gray bars) was determined after 90 min of infection. Gentamycin was added to medium for another 90 min (total of 180 min) to assess the invasion level (black bars). **(A)** Mutant strains with deletion of each fimbrial gene cluster were constructed and used for this assay. **(B)** Complete fimbrial gene cluster (including native promoter) was cloned on low-copy pWSK29 vector and transformed into afimbrial ISP1820. Results are expressed as the mean ± SEM of at least three distinct experiments performed in triplicates. ^*^p < 0.05; ^**^p < 0.01; ^***^p < 0.001.

The role of fimbriae in adhesion to or invasion of intestinal epithelial INT-407 cells was evaluated. First, the effect of the deletion of a single fimbrial system was tested (Figure 5A). Deletion of *fim* and *sef* provoked a decrease in adherence. However, every fimbrial deletion caused a decrease in invasion to epithelial cells, only Δ*bcf* and Δ*ste* mutants were not significant when compared to the wild-type. The addition of a single fimbrial system (under its native promoter) in the afimbrial strain (Figure 5B) was then tested. The addition of Fim, Saf, Sef, Stb, Stc, or Ste caused a significant decrease in adherence and addition of any fimbrial system provoked a decrease in invasion of epithelial cells. Overall, fimbriae seem to have a generalized deleterious effect on invasion of epithelial cells, but variable effects on adherence.

### Uptake and survival within macrophages

**Figure 6:**
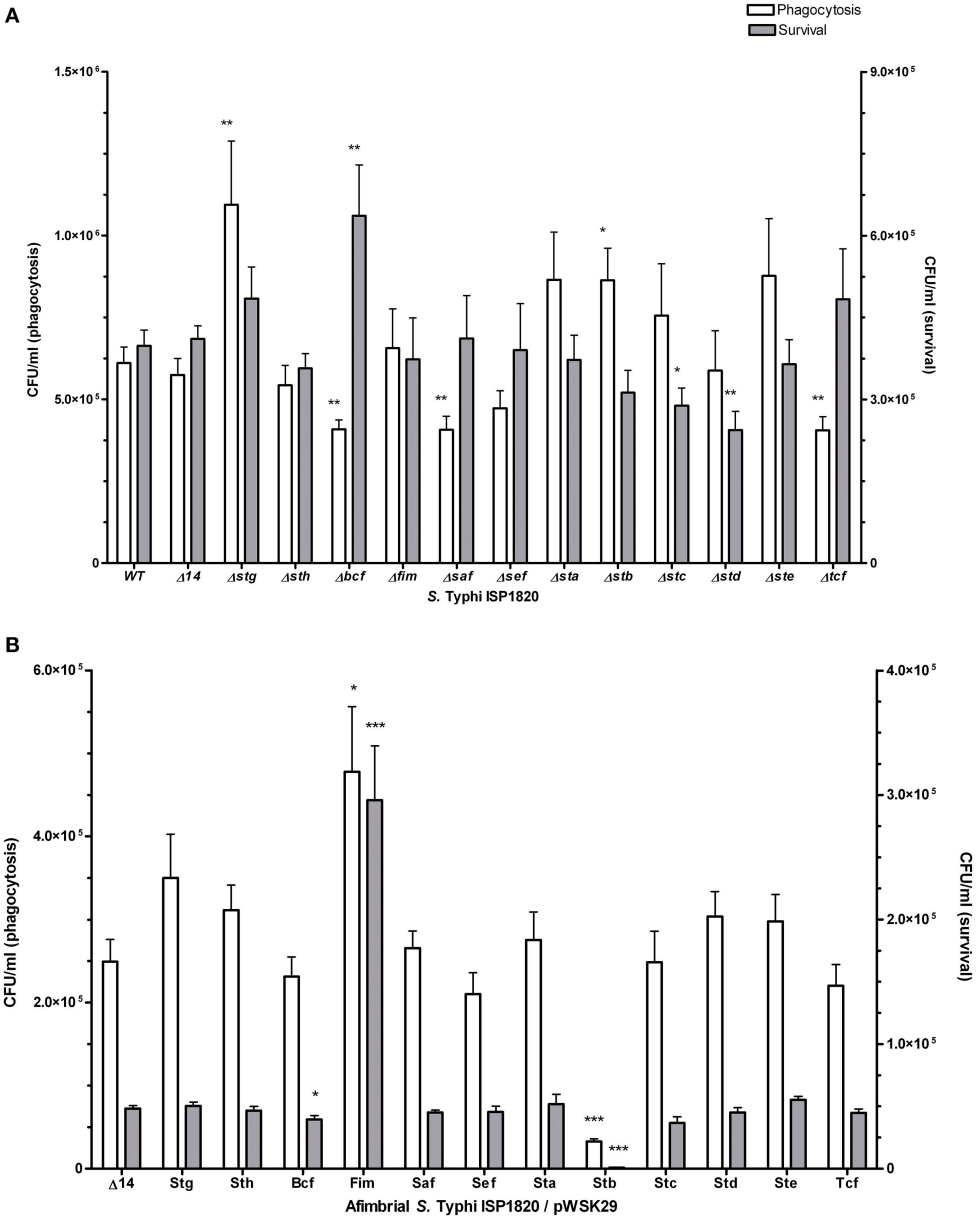
Fimbrial interactions with THP-1 macrophages. Phagocytosis (white bars) was measured after 20 min of infection. Gentamycin was added to medium overnight to assess the survival (gray bars). **(A)** Mutant strains with deletion of each fimbrial gene cluster were used for this assay. **(B)** Native promoter and operon was cloned on low-copy pWSK29 vector and transformed into afimbrial ISP1820. Results are expressed as the mean ± SEM of at least three distinct experiments performed in triplicates. ^*^p < 0.05; ^**^p < 0.01; ^***^p < 0.001.

Interaction of *S*. Typhi with THP-1 macrophages was assessed for phagocytosis (*t* = 0) and for survival after 24 h (Figure 6A). Deletion of *stg* and *stb* significantly increased phagocytosis levels compared to the wild-type (up to 160 and 155%, respectively) while deletion of *bcf*, *saf*, and *tcf* decreased it to 88, 65, and 87% respectively. Deletion of *bcf* increased survival to 126% of the wild-type strain, whereas deletion of *stc* and *std* resulted in decreased survival to 68 and 48% respectively compared to the wild-type strain. Regarding the addition of individual fimbria to the afimbrial strain, Fim and Stb present the most relevant phenotypes (Figure 6B): Fim fimbriae increased phagocytosis (259%) and survival (600%), whereas Stb decreased phagocytosis (10%) and survival (2%).

### Fimbriae and motility

The role of fimbriae on motility was tested on LB 0.3% agar plates. Either the deletion of one fimbrial system or the addition of individual fimbrial systems to the afimbrial strain did not show any significant phenotype compared to the wild-type control, as the same level of swimming motility was observed (data not shown). However, when each fimbrial system was overexpressed, the Fim fimbriae (pMMBfim) drastically decreased the swimming to 52.3 ± 3.2% of the control. By contrast, none of the other fimbrial systems showed a significant difference when compared to the control strain (data not shown).

### Role of fimbriae on biofilm production

**Figure 7:**
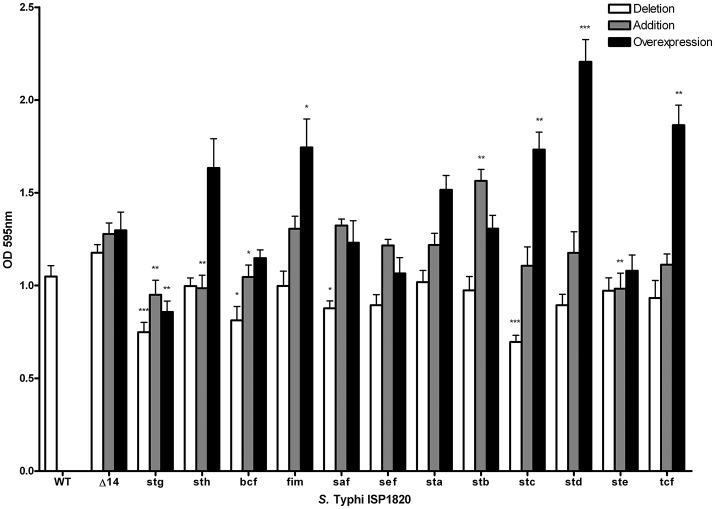
Impact of fimbriae on biofilm formation. Biofilm formation was performed with cholesterol-coated plate and bacteria were incubated statically for 72 h in a bile-supplemented medium. Results are expressed as the mean ± SEM of at least three distinct experiments performed in triplicates. White bars: Mutant strains with deletion of each fimbrial gene cluster were used for this assay. Gray bars: Native promoter and operon was cloned on low-copy pWSK29 vector and transformed into afimbrial ISP1820. Black bars: Native operon was cloned under lactose-inducible promoter (p_TAC_) on pMMB207c vector and transformed into afimbrial ISP1820. ^*^p < 0.05; ^**^p < 0.01; ^***^p < 0.001.

The role of fimbriae for biofilm formation was tested by crystal violet coloration assays. First, the consequence of the deletion of individual fimbrial systems was evaluated (Figure 7). Deletion of *stg, bcf*, *saf* or *stc* decreased biofilm production to between 70 and 85% of the wild-type production. Introduction of each the individual fimbrial systems to the afimbrial strain was also evaluated (Figure 7). Addition of Stg, Sth, Bcf, and Ste reduced biofilm production to 72–88% of the control and addition of Stb increased biofilm production to 128%. We then tested the effect of fimbrial overexpression by induction of the fimbrial cluster (Figure 7). Fim, Stc, Std, and Tcf increased biofilm production to levels between 140 and 180% of the control strain, whereas Stg had decreased biofilm production to 68%.

## Discussion

*S*. Typhi possesses 12 different chaperone/usher fimbriae that belong to 5 fimbrial clades (γ_1_, γ_3_, γ_4_, π, and α) based on usher homologies (Nuccio and Bäumler, 2007). These fimbriae were identified by whole-genome sequencing and most are considered to be putative, as it is not known under what conditions they may be expressed or they have not been characterized or visualized yet (Baker and Dougan, 2007). Here, we investigated the expression and characterization of all 12 of the *S*. Typhi CU fimbriae. We evaluated the expression of fimbrial promoters, production of fimbrial proteins and functional production of fimbriae. We also characterized the effects of fimbrial deletions or phenotypes due to introduction of specific fimbrial gene clusters to an afimbrial strain on interaction with host epithelial cells and macrophages, bacterial motility, and biofilm formation.

Fimbriae are poorly expressed when grown under laboratory conditions. A study from Kröger et al. (2013) compared 22 infection-mimicking conditions by an RNA-seq-based analysis of *S*. Typhimurium SL1344 and revealed that fimbrial genes, including the 7 CU fimbrial operons in common with *S*. Typhi, were not or poorly expressed. To determine the expression of *S*. Typhi CU fimbrial promoters, the use of a multi-copy reporter gene fusion (pRS415) with each fimbrial promoter was required to obtain a sufficient β-galactosidase activity, as a chromosomal *lacZ* fusion did not provide a sufficient level of expression for quantification of Miller units (data not shown). Six growth conditions were tested: rich (LB), minimal (M63), both in liquid (broth) and solid (agar), and SPI-1 and−2 T3SSs induction conditions (Figure 2). As the niche of *S*. Typhi is restricted to the human body, only 37°C was tested. Interestingly, one specific fimbrial promoter from each of the different CU clades was dominant (*sth* for γ_1_, *saf* for γ_3_, *stc* for γ_4_, and *std* for π), suggesting a role for each fimbrial clade in *S*. Typhi. Also, the majority of the fimbrial systems had a unique expression pattern, except for *stg* and *ste* (Table 1). The highest expression for each fimbrial promoter was obtained during growth in minimal medium. Low nutrient conditions may be encountered by *S*. Typhi during colonization of liver and spleen, in the blood, or in the gall bladder, corresponding to different sites where fimbriae may be needed (Gonzalez-Escobedo et al., 2011; Keestra-Gounder et al., 2015). SPI-1 T3SS-inducing condition was usually the condition with the lowest expression, except for *stc* and *std* which demonstrated their lowest on M63 agar. In *S*. Typhimurium, the balance between adherence (fimbriae), invasion (SPI-1 T3SS) and motility (flagella) is usually finely regulated. The induction of one of these elements can therefore result in a decrease in expression of the other factors (Clegg and Hughes, 2002; Saini et al., 2010; Baxter and Jones, 2015). Otherwise, other SPI-1 inducing conditions might be more optimal for the expression of the studied fimbriae and might reflect more accurately interaction with epithelial cells.

**Table 1:** Summary of fimbrial expression and pathogenesis phenotypes.

**Fimbriae**	**Promoter expression**						**INT**−**407 infection**				**THP**−**1 infection**				**Motility**	**Biofilm formation**		
							**Adherence**		**Invasion**		**Uptake**		**Survival**					
	**M63 broth**	**M63 agar**	**LB broth**	**LB agar**	**SPI−1**	**SPI−2**	**Deletion**	**Addition**	**Deletion**	**Addition**	**Deletion**	**Addition**	**Deletion**	**Addition**	**Overexpression**	**Deletion**	**Addition**	**Overexpression**
**Stg**	**1**	**5**	**2**	**3**	**6**	**4**	0	0	−	−	+	0	0	0	0	−	−	−
**Sth**	**1**	**4**	**3**	**2**	**6**	**5**	0	0	−	−	0	0	0	0	0	0	−	0
**Bcf**	**1**	**3**	**4**	**2**	**6**	**5**	0	0	0	−	−	0	+	−	0	−	−	0
**Fim**	**1**	**3**	**2**	**4**	**6**	**5**	−	−	−	−	0	+	0	+	−	0	0	+
**Saf**	**1**	**5**	**2**	**4**	**6**	**3**	0	−	−	−	−	0	0	0	0	−	0	0
**Sef**	**1**	**4**	**3**	**5**	**6**	**2**	−	−	−	−	0	0	0	0	0	0	0	0
**Sta**	**1**	**5**	**2**	**4**	**6**	**3**	0	0	−	−	0	0	0	0	0	0	0	0
**Stb**	**1**	**3**	**2**	**5**	**6**	**4**	0	−	−	−	+	−	0	−	0	0	+	0
**Stc**	**1**	**6**	**2**	**5**	**4**	**3**	0	−	−	−	0	0	−	0	0	−	0	+
**Std**	**1**	**6**	**2**	**3**	**4**	**5**	0	0	−	−	0	0	−	0	0	0	0	+
**Ste**	**1**	**5**	**2**	**3**	**6**	**4**	0	−	0	−	0	0	0	0	0	0	−	0
**Tcf**	**1**	**5**	**3**	**4**	**6**	**2**	0	0	−	−	−	0	0	0	0	0	0	+

*SPI-1, SPI−1 T3SS induction; SPI-2, SPI−2 T3SS induction; Deletion, mutant strains; Addition, afimbrial strain with pWSK29 vector; Overexpression, afimbrial strain with pMMB207c vector; 1–6, descending order of expression; 0, no effect; –, deleterious effect; +, increasing effect*.

As fimbriae are poorly expressed in laboratory conditions, the functional assembly of putative fimbrial operons of *S*. Typhi was induced and detection of the major subunit proteins were visualized for Fim, Saf, Sta, Stb, Stc, Std, and Tcf systems. No specific bands were observed for putative fimbrial gene clusters that contain pseudogenes. This could be explained by lack of functional assembly for these fimbriae. Fim, Saf, Sta, Stb, Std, and Tcf fimbriae were also visualized by TEM and demonstrated differences in morphology and distribution on the surface of cells which may imply difference in pathogenesis functions (Figure 4). Fimbrial gene clusters with pseudogenes (*stg, sth, bcf*, *sef*, and *ste*) and Stc fimbriae were not observed by TEM. This can be due to their detachment during the grid preparation or to the absence of functional assembly. Most of the pseudogenes are located in usher or subunits genes that may prevent the formation of the fimbriae by avoiding the transport of the subunits through the outer-membrane or the fimbriae formation. However, fimbrial operons with pseudogenes may be functional by complementing the non-functional usher by another fimbrial usher or by using a suppressor tRNAs to bypass stop codon to form a functional usher or subunit (Berrocal et al., 2015). The presence of pseudogenes may still impact on pathogenesis as some of the fimbrial proteins may be exported in the environment or fixed to the surface in absence of full-length fimbriae, acting similarly to afimbrial adhesin.

The potential roles of the different CU fimbriae were investigated by testing effects of the individual deletion of these systems from the wild-type strain or by introduction of each system to an afimbrial *S*. Typhi strain on bacterial interactions with host cells (INT-407 and THP-1) (Figures 5–6, Table 1), on motility, and on biofilm formation (Figure 7). Interestingly, each fimbria seems to have a role in invasion as deletion of one of them or individual addition of fimbriae in afimbrial strain decrease invasion to epithelial cells compared to their control. This implies that fimbriae may affect invasion by regulation of the SPI-1 T3SS or that they are critical for the initial contact with host cells to allow a stable interaction between the T3SS and the host. A combination of multiple fimbriae may be important for the optimal contact between the bacteria and the epithelial cells. Another hypothesis is that the SPI-1 T3SS or other adhesins at the surface of the bacteria are affected by the deletion of fimbriae and may confer lower invasion of epithelial cells. The functionality of the T3SS may be verified by a secretion assay in further studies and the presence of different adhesins could be determined by Western Blot. There was no difference in motility, except for induction of Fim, whereas most of fimbriae had variable effects (positive or negative) on biofilm formation, except for Sef and Sta. Specific results for each fimbrial system are discussed in the next sections.

The highest expressed promoter was *saf*. Saf fimbriae were also visualized on TEM. Those fimbriae are conserved among *S. enterica* subspecies *enterica* (ssp. I). No noticeable phenotype during cell infection, motility or biofilm formation has been previously reported (Folkesson et al., 1999; Humphries et al., 2003). However, *S*. Typhi Saf fimbriae were strongly expressed inside human macrophages (Faucher et al., 2006). The main effect observed for the *saf* deletion mutant of ISP1820 was decreased phagocytosis, invasion and biofilm formation (Table 1). Also, the addition of the *saf* gene cluster to the afimbrial strain decreased adherence and invasion to epithelial cells. In summary, Saf fimbriae are the most expressed fimbriae in *S*. Typhi in conditions tested, demonstrated specificity for macrophages and may be implicated in biofilm formation.

Std and Stc are highly expressed and the only systems with an expression level in SPI-1 T3SS inducing conditions that was not their lowest (Table 1). They also share similarities in infection of host cells, with a decrease of survival in macrophages, when expressed, and in biofilm formation when overexpressed. Std and Stc fimbriae are widely distributed in *Salmonella enterica* ssp. I, forming the core fimbriae with Csg (curli fimbriae), Fim, Sth, Bcf, and Stb (Dufresne, 2017). Interestingly, *S*. Typhimurium both Std and Stc fimbriae were recognized to be involved in intestinal persistence in mice (Weening et al., 2005). The role of these two fimbriae for invasion and survival could be generalized to other serovars of *S. enterica* ssp. I and their affinity could be for the intestinal epithelial cells.

*sth* demonstrated a high level of expression similar to levels of *stc* expression and is highly expressed when grown on LB agar, similarly to *bcf*, which could suggest a role for these fimbriae on solid surfaces (Table 1). However, Sth did not contribute significantly to adhesion to or interaction with human cells, whereas the deletion of *bcf* caused a decrease in phagocytosis, but an increase in survival in macrophages. Addition of *sth* or *bcf* gene clusters reduced biofilm formation, suggesting that their role is not in interbacterial adhesion but may be important in adherence to other host cells, such as splenocytes, hepatocytes, or cells from the gall bladder, or in environmental conditions. These two fimbriae are present in most serovars of *S. enterica* ssp. I, and also belong to the fimbrial core. However, as *S*. Typhi is a pathogen with a restricted niche, the presence of pseudogenes in those two gene clusters may partially explain why the bacterium is human-specific. Also, the truncated ushers may allow the export of fimbrial proteins in extracellular environment, which may explain the effects on pathogenesis presented by Sth and Bcf fimbriae.

*stg* and *ste* promoters exhibited the same pattern of expression but not at same level, *stg* expression was moderate whereas *ste* expression was lower (Table 1). Deletion of *stg* from *S*. Typhi ISP1820 increased phagocytosis in macrophages and is consistent with Forest et al. (2007) as they used *S*. Typhi ISP1820 and THP-1 macrophages as well. Berrocal et al. (2015) however obtained results that loss of *stg* decreased phagocytosis, but they used *S*. Typhi STH2370, a Chilean isolate, and a different macrophage cell line. Stg has deleterious effects on biofilm formation, regardless of its deletion, addition or overexpression. The deleterious effect of the deletion of *stg* suggests that this fimbria is involved in biofilm formation. However, the effect of addition or overexpression of Stg in the afimbrial strain suggests that other fimbriae must modulate action of Stg and are required for establishing a mature biofilm. Addition of the Ste fimbriae also reduced biofilm formation. These two fimbriae have pseudogenes in their operon and are not present in *S*. Typhimurium. Despite the presence of pseudogenes and the absence of fimbrial assembly suggesting these gene clusters may not be functional, these fimbrial systems contribute during bacterial interactions with host cells and biofilm formation.

Fim and Stb fimbriae had poor expression in any growth conditions tested. However, they showed major phenotypes in most of the assays. Deletion of *fim* decreased adherence and invasion to epithelial cells, while addition of Fim to the afimbrial strain also decreased adherence and invasion to INT-407 cells. When added to the afimbrial strain, Fim increased phagocytosis and survival in macrophages. Different studies involved FimH, the adhesin of Fim fimbriae, as an important marker of host-specificity. For *S*. Typhi, the presence of a valine in position 223 of FimH seems to be determinant for the binding of human cell lines (Kisiela et al., 2012; Yue et al., 2015). The defect in swimming may be due by the decrease in growth rate or by overexpression of the Fim fimbriae. Fim fimbriae may be a major player of *S*. Typhi pathogenesis and may regulate other fimbriae and virulence factors like flagella, LPS or T3SSs. Stb may be involved in long-term persistence by biofilm formation and may regulate negatively early stages of infection like host cell interactions. The balance between motility, invasion and persistence is critical for a successful infection, and Stb seems to inhibit invasion in favor of persistence. The inhibition of interaction with macrophages by Stb fimbriae could be physical: Stb fimbriae are long and cover the majority of the surface of the bacteria, which may interfere with recognition molecules for receptors on macrophages, and influence the interaction with host cells. Stb and Fim may have opposing roles on macrophages/bacteria interaction and, in a wild-type strain, may counteract the action of each other at different steps to lead to a successful infection.

Expression of the *tcf* promoter was low in every condition tested. However, Tcf fimbriae were previously visualized and are similar to cable (Cbl) pili of *Burkholderia cepacia*, which are in the same CU clade (α) and share more genetic homologies than other members of the α clade (Leclerc et al., 2016). Deletion of *tcf* decreased phagocytosis by macrophages, but does not affect survival. Overexpression of Tcf fimbriae increased biofilm formation. Tcf may play a role in late stages of infection, from the interaction with macrophages to persistence in gall bladder.

Deletion of the *sef* gene cluster decreases adherence and invasion of epithelial cells. However, its presence in an afimbrial strain did not allow recovery of the control levels. Despite the presence of a pseudogene in its operon, *sef* influenced epithelial cell interactions.

Overall, CU fimbriae act as redundant systems on different aspects of *S*. Typhi pathogenesis, such as bacteria/host cell interactions and biofilm formation. In order to reflect more accurately the *S*. Typhi pathogenesis, fimbriae should be also studied in interactions with other host cells targets such as splenocytes, hepatocytes, and cells from the gall bladder. Some fimbriae are similar in their functions and usually have reverse level of expression in a matter of balanced expression (Supplementary Figure 1). The similarity between certain fimbriae could be a form of functional redundancy. Fimbrial production is energetically demanding for the bacteria and the balance of expression between few fimbriae of similar function and presence of pseudogenes could be a way to prevent unnecessary redundancy and preserve *S*. Typhi energy. A tightly regulated equilibrium between stages of pathogenicity of *S*. Typhi is important for a successful infection of the host. The presence of fimbriae at the surface of the bacteria is confirmed for Fim, Saf, Sta, Stb, Std, and Tcf. Each of these fimbriae affect invasion of epithelial cells and phagocytosis by macrophages, although Std only affected survival in macrophages. Fimbriae such as Stc and gene clusters containing pseudogenes (*bcf*, *stg, ste*, and *sef*) also contributed to different aspects of infection based on our investigation. Each of these systems had an impact on interactions with epithelial cells, although *sef* and *ste* did not alter macrophage interactions. Further, they all had an effect on biofilm formation, except for *sef* and *sta*. Sta and Sth do not present particular function in the case of *S*. Typhi. Sta could be involved in a process of human-specificity, as it is only present in host-restricted strains. For Sth, it may lose its function in *S*. Typhi as it could have an importance in adhesion to abiotic surfaces in other serovars. Fimbriae are critical components of the equilibrium between stages of pathogenesis and further research is needed to more fully understand their complex role for *S*. Typhi and the pathogenesis of typhoid fever.

## Authors contributions

KD and FD: Designed the research; KD and JS-B: Proceed to the experiments; KD and FD: Analyzed the data; KD: Drafted the manuscript; KD, FD, and JS-B: Revised the manuscript.

### Conflict of interest statement

The authors declare that the research was conducted in the absence of any commercial or financial relationships that could be construed as a potential conflict of interest.

## References

[B1] Baker S. Dougan G. (2007). The genome of *Salmonella enterica* serovar Typhi. Clin. Infect. Dis. 45(Suppl. 1), S29–S33. 10.1086/518143 17582565

[B2] Baxter M. A. Jones B. D. (2015). Two-component regulators control hilA expression by controlling fimZ and hilE expression within *Salmonella enterica* serovar Typhimurium. Infect. Immun. 83, 978–985. 10.1128/IAI.02506-14 25547794 PMC4333451

[B3] Beloin C. Michaelis K. Lindner K. Landini P. Hacker J. Ghigo J. M.. (2006). The transcriptional antiterminator RfaH represses biofilm formation in *Escherichia coli*. J. Bacteriol. 188, 1316–1331. 10.1128/JB.188.4.1316-1331.2006 16452414 PMC1367212

[B4] Berrocal L. Fuentes J. A. Trombert A. N. Jofré M. R. Villagra N. A. Valenzuela L. M.. (2015). *stg* fimbrial operon from *S*. Typhi STH2370 contributes to association and cell disruption of epithelial and macrophage-like cells. Biol. Res. 48:34. 10.1186/s40659-015-0024-9 26149381 PMC4494162

[B5] Bishop A. House D. Perkins T. Baker S. Kingsley R. A. Dougan G. (2008). Interaction of *Salmonella enterica* serovar Typhi with cultured epithelial cells: roles of surface structures in adhesion and invasion. Microbiology 154(Pt 7), 1914–1926. 10.1099/mic.0.2008/016998-0 18599820 PMC2652038

[B6] Boddicker J. D. Ledeboer N. A. Jagnow J. Jones B. D. Clegg S. (2002). Differential binding to and biofilm formation on, HEp-2 cells by *Salmonella enterica* serovar Typhimurium is dependent upon allelic variation in the *fimH* gene of the *fim* gene cluster. Mol. Microbiol. 45, 1255–1265. 10.1046/j.1365-2958.2002.03121.x 12207694

[B7] Chang S. J. Song J. Galán J. E. (2016). Receptor-Mediated sorting of typhoid toxin during its export from *Salmonella* typhi-infected cells. Cell Host Microbe 20, 682–689. 10.1016/j.chom.2016.10.005 27832592 PMC5110213

[B8] Clark M. A. Jepson M. A. Simmons N. L. Hirst B. H. (1994). Preferential interaction of *Salmonella* typhimurium with mouse Peyer's patch M cells. Res. Microbiol. 145, 543–552. 10.1016/0923-2508(94)90031-0 7855440

[B9] Clegg S. Hughes K. T. (2002). FimZ is a molecular link between sticking and swimming in *Salmonella enterica* serovar Typhimurium. J. Bacteriol. 184, 1209–1213. 10.1128/jb.184.4.1209-1213.2002 11807085 PMC134799

[B10] Coombes B. K. Brown N. F. Valdez Y. Brumell J. H. Finlay B. B. (2004). Expression and secretion of *Salmonella* pathogenicity island-2 virulence genes in response to acidification exhibit differential requirements of a functional type III secretion apparatus and SsaL. J. Biol. Chem. 279, 49804–49815. 10.1074/jbc.M404299200 15383528

[B11] Daigle F. Graham J. E. Curtiss R. III. (2001). Identification of *Salmonella* typhi genes expressed within macrophages by selective capture of transcribed sequences (SCOTS). Mol. Microbiol. 41, 1211–1222. 10.1046/j.1365-2958.2001.02593.x 11555299

[B12] Datsenko K. A. Wanner B. L. (2000). One-step inactivation of chromosomal genes in *Escherichia coli* K-12 using PCR products. Proc. Natl. Acad. Sci. U.S.A. 97, 6640–6645. 10.1073/pnas.120163297 10829079 PMC18686

[B13] De Masi L. Yue M. Hu C. Rakov A. V. Rankin S. C. Schifferli D. M. (2017). Cooperation of adhesin alleles in *Salmonella*-Host Tropism. mSphere 2:e00066–17. 10.1128/mSphere.00066-17 28289725 PMC5343171

[B14] Dufresne K. D. F. (2017). Salmonella Fimbriae: what is the clue to their hairdo? in Current Topics in Salmonella and Salmonellosis, ed Mares M. (Rijeka: InTech.), 59–79.

[B15] Edwards R. A. Schifferli D. M. Maloy S. R. (2000). A role for *Salmonella* fimbriae in intraperitoneal infections. Proc. Natl. Acad. Sci. U.S.A. 97, 1258–1262. 10.1073/pnas.97.3.1258 10655518 PMC15588

[B16] Elhadad D. Desai P. Grassl G. A. McClelland M. Rahav G. Gal-Mor O. (2016). Differences in host cell invasion and *Salmonella* Pathogenicity Island 1 expression between *Salmonella enterica* Serovar paratyphi A and nontyphoidal S. Typhimurium. Infect. Immun. 84, 1150–1165. 10.1128/IAI.01461-15 26857569 PMC4807488

[B17] Faucher S. P. Porwollik S. Dozois C. M. McClelland M. Daigle F. (2006). Transcriptome of *Salmonella enterica* serovar Typhi within macrophages revealed through the selective capture of transcribed sequences. Proc. Natl. Acad. Sci. U.S.A. 103, 1906–1911. 10.1073/pnas.0509183103 16443683 PMC1413645

[B18] Folkesson A. Advani A. Sukupolvi S. Pfeifer J. D. Normark S. Löfdahl S. (1999). Multiple insertions of fimbrial operons correlate with the evolution of *Salmonella* serovars responsible for human disease. Mol. Microbiol. 33, 612–622. 10.1046/j.1365-2958.1999.01508.x 10417651

[B19] Forest C. Faucher S. P. Poirier K. Houle S. Dozois C. M. Daigle F. (2007). Contribution of the *stg* fimbrial operon of *Salmonella enterica* serovar Typhi during interaction with human cells. Infect. Immun. 75, 5264–5271. 10.1128/IAI.00674-07 17709421 PMC2168283

[B20] Galán J. E. (2001). *Salmonella* interactions with host cells: type III secretion at work. Annu. Rev. Cell Dev. Biol. 17, 53–86. 10.1146/annurev.cellbio.17.1.53 11687484

[B21] Galan J. E. Zhou D. (2000). Striking a balance: modulation of the actin cytoskeleton by *Salmonella*. Proc. Natl. Acad. Sci. U.S.A. 97, 8754–8761. 10.1073/pnas.97.16.8754 10922031 PMC34008

[B22] Ganjali Dashti M. Abdeshahian P. Sudesh K. Phua K. K. (2016). Optimization of *Salmonella* Typhi biofilm assay on polypropylene microtiter plates using response surface methodology. Biofouling 32, 477–487. 10.1080/08927014.2015.1135328 26963754

[B23] Gonzales A. M. Wilde S. Roland K. L. (2017). New insights into the roles of long polar fimbriae and Stg fimbriae in *Salmonella* interactions with enterocytes and M cells. Infect. Immun. 85:e00172–17 10.1128/IAI.00172-17 28630073 PMC5563581

[B24] Gonzalez-Escobedo G. Marshall J. M. Gunn J. S. (2011). Chronic and acute infection of the gall bladder by *Salmonella* Typhi: understanding the carrier state. Nat. Rev. Microbiol. 9, 9–14. 10.1038/nrmicro2490 21113180 PMC3255095

[B25] Horstmann J. A. Zschieschang E. Truschel T. de Diego J. Lunelli M. Rohde M.. (2017). Flagellin phase-dependent swimming on epithelial cell surfaces contributes to productive *Salmonella* gut colonisation. Cell. Microbiol. 19:e12739. 10.1111/cmi.12739 28295924

[B26] Humphries A. D. Raffatellu M. Winter S. Weening E. H. Kingsley R. A. Droleskey R.. (2003). The use of flow cytometry to detect expression of subunits encoded by 11 *Salmonella enterica* serotype Typhimurium fimbrial operons. Mol. Microbiol. 48, 1357–1376. 10.1046/j.1365-2958.2003.03507.x 12787362

[B27] Jiang L. Feng L. Yang B. Zhang W. Wang P. Jiang X.. (2017). Signal transduction pathway mediated by the novel regulator LoiA for low oxygen tension induced *Salmonella* Typhimurium invasion. PLoS Pathog. 13:e1006429. 10.1371/journal.ppat.1006429 28575106 PMC5476282

[B28] Kaniga K. Delor I. Cornelis G. R. (1991). A wide-host-range suicide vector for improving reverse genetics in gram-negative bacteria: inactivation of the *blaA* gene of *Yersinia enterocolitica*. Gene 109, 137–141. 10.1016/0378-1119(91)90599-7 1756974

[B29] Keestra-Gounder A. M. Tsolis R. M. Bäumler A. J. (2015). Now you see me, now you don't: the interaction of *Salmonella* with innate immune receptors. Nat. Rev. Microbiol. 13, 206–216. 10.1038/nrmicro3428 25749454

[B30] Kisiela D. I. Chattopadhyay S. Libby S. J. Karlinsey J. E. Fang F. C. Tchesnokova V.. (2012). Evolution of *Salmonella enterica* virulence via point mutations in the fimbrial adhesin. PLoS Pathog. 8:e1002733. 10.1371/journal.ppat.1002733 22685400 PMC3369946

[B31] Kröger C. Colgan A. Srikumar S. Handler K. Sivasankaran S. K. Hammarlof D. L.. (2013). An infection-relevant transcriptomic compendium for *Salmonella enterica* Serovar Typhimurium. Cell Host Microbe 14, 683–695. 10.1016/j.chom.2013.11.010 24331466

[B32] Leclerc J. M. Dozois C. M. Daigle F. (2013). Role of the *Salmonella enterica* serovar Typhi Fur regulator and small RNAs RfrA and RfrB in iron homeostasis and interaction with host cells. Microbiology 159(Pt 3), 591–602. 10.1099/mic.0.064329-0 23306672

[B33] Leclerc J. M. Quevillon E. L. Houde Y. Paranjape K. Dozois C. M. Daigle F. (2016). Regulation and production of Tcf, a cable-like fimbriae from *Salmonella enterica* serovar Typhi. Microbiology 162, 777–788. 10.1099/mic.0.000270 26944792

[B34] Ledeboer N. A. Frye J. G. McClelland M. Jones B. D. (2006). *Salmonella enterica* serovar Typhimurium requires the Lpf, Pef, and Tafi fimbriae for biofilm formation on HEp-2 tissue culture cells and chicken intestinal epithelium. Infect. Immun. 74, 3156–3169. 10.1128/IAI.01428-05 16714543 PMC1479237

[B35] Lee C. A. Jones B. D. Falkow S. (1992). Identification of a *Salmonella* typhimurium invasion locus by selection for hyperinvasive mutants. Proc. Natl. Acad. Sci. U.S.A. 89, 1847–1851. 10.1073/pnas.89.5.1847 1311853 PMC48550

[B36] Low A. S. Dziva F. Torres A. G. Martinez J. L. Rosser T. Naylor S.. (2006). Cloning, expression, and characterization of fimbrial operon F9 from enterohemorrhagic *Escherichia coli* O157:H7. Infect. Immun. 74, 2233–2244. 10.1128/IAI.74.4.2233-2244.2006 16552054 PMC1418889

[B37] Miller J. H. (1972). Experiments in Molecular Genetics. Cold Spring Harbor, NY: Cold Spring Harbor Laboratory.

[B38] Morales V. M. Bäckman A. Bagdasarian M. (1991). A series of wide-host-range low-copy-number vectors that allow direct screening for recombinants. Gene 97, 39–47. 10.1016/0378-1119(91)90007-X 1847347

[B39] Nuccio S. P. Bäumler A. J. (2007). Evolution of the chaperone/usher assembly pathway: fimbrial classification goes Greek. Microbiol. Mol. Biol. Rev. 71, 551–575. 10.1128/MMBR.00014-07 18063717 PMC2168650

[B40] Obaro S. K. Iroh Tam P. Y. Mintz E. D. (2017). The unrecognized burden of typhoid fever. Expert Rev. Vaccines 16, 249–260. 10.1080/14760584.2017.1255553 27797598

[B41] O'Callaghan D. Charbit A. (1990). High efficiency transformation of *Salmonella* typhimurium and *Salmonella* typhi by electroporation. Mol. Gen. Genet. 223, 156–158. 10.1007/BF00315809 2259337

[B42] Pokharel B. M. Koirala J. Dahal R. K. Mishra S. K. Khadga P. K. Tuladhar N. R. (2006). Multidrug-resistant and extended-spectrum beta-lactamase (ESBL)-producing *Salmonella enterica* (serotypes Typhi and Paratyphi A) from blood isolates in Nepal: surveillance of resistance and a search for newer alternatives. Int. J. Infect. Dis. 10, 434–438. 10.1016/j.ijid.2006.07.001 16978898

[B43] Qamar F. N. Azmatullah A. Bhutta Z. A. (2015). Challenges in measuring complications and death due to invasive *Salmonella* infections. Vaccine 33 (Suppl. 3), C16–C20. 10.1016/j.vaccine.2015.03.103 25921727

[B44] Rowe B. Ward L. R. Threlfall E. J. (1997). Multidrug-resistant *Salmonella* Typhi: a worldwide epidemic. Clin. Infect. Dis. 24 (Suppl. 1), S106–109. 10.1093/clinids/24.Supplement_1.S106 8994789

[B45] Sabbagh S. C. Lepage C. McClelland M. Daigle F. (2012). Selection of *Salmonella enterica* serovar Typhi genes involved during interaction with human macrophages by screening of a transposon mutant library. PLoS ONE 7:e36643. 10.1371/journal.pone.0036643 22574205 PMC3344905

[B46] Saini S. Slauch J. M. Aldridge P. D. Rao C. V. (2010). Role of cross talk in regulating the dynamic expression of the flagellar *Salmonella* pathogenicity island 1 and type 1 fimbrial genes. J. Bacteriol. 192, 5767–5777. 10.1128/JB.00624-10 20833811 PMC2953706

[B47] Thong K. L. Bhutta Z. A. Pang T. (2000). Multidrug-resistant strains of *Salmonella enterica* serotype typhi are genetically homogenous and coexist with antibiotic-sensitive strains as distinct, independent clones. Int. J. Infect. Dis. 4, 194–197. 10.1016/S1201-9712(00)90108-5 11231181

[B48] Townsend S. M. Kramer N. E. Edwards R. Baker S. Hamlin N. Simmonds M.. (2001). *Salmonella enterica* serovar Typhi possesses a unique repertoire of fimbrial gene sequences. Infect. Immun. 69, 2894–2901. 10.1128/IAI.69.5.2894-2901.2001 11292704 PMC98240

[B49] Tremblay Y. D. Vogeleer P. Jacques M. Harel J. (2015). High-throughput microfluidic method to study biofilm formation and host-pathogen interactions in pathogenic *Escherichia coli*. Appl. Environ. Microbiol. 81, 2827–2840. 10.1128/AEM.04208-14 25681176 PMC4375333

[B50] Tsui I. S. Yip C. M. Hackett J. Morris C. (2003). The type IVB pili of *Salmonella enterica* serovar Typhi bind to the cystic fibrosis transmembrane conductance regulator. Infect. Immun. 71, 6049–6050. 10.1128/IAI.71.10.6049-6050.2003 14500527 PMC201034

[B51] Wang R. F. Kushner S. R. (1991). Construction of versatile low-copy-number vectors for cloning, sequencing and gene expression in *Escherichia coli*. Gene 100, 195–199. 10.1016/0378-1119(91)90366-J 2055470

[B52] Wangdi T. Lee C. Y. Spees A. M. Yu C. Kingsbury D. D. Winter S. E.. (2014). The Vi capsular polysaccharide enables *Salmonella enterica* serovar typhi to evade microbe-guided neutrophil chemotaxis. PLoS Pathog. 10:e1004306. 10.1371/journal.ppat.1004306 25101794 PMC4125291

[B53] Waterman S. R. Holden D. W. (2003). Functions and effectors of the *Salmonella* pathogenicity island 2 type III secretion system. Cell. Microbiol. 5, 501–511. 10.1046/j.1462-5822.2003.00294.x 12864810

[B54] Weening E. H. Barker J. D. Laarakker M. C. Humphries A. D. Tsolis R. M. Bäumler A. J. (2005). The *Salmonella enterica* serotype Typhimurium *lpf, bcf*, *stb, stc, std*, and *sth* fimbrial operons are required for intestinal persistence in mice. Infect. Immun. 73, 3358–3366. 10.1128/IAI.73.6.3358-3366.2005 15908362 PMC1111867

[B55] Weinstein D. L. O'Neill B. L. Hone D. M. Metcalf E. S. (1998). Differential early interactions between *Salmonella enterica* serovar Typhi and two other pathogenic *Salmonella* serovars with intestinal epithelial cells. Infect. Immun. 66, 2310–2318. 9573122 10.1128/iai.66.5.2310-2318.1998 PMC108196

[B56] WHO (2012). Report of the Ad-hoc Consultation on Typhoid Vaccine Introduction and Typhoid Surveillance. Geneva: World Health Organization.

[B57] Wilson R. P. Raffatellu M. Chessa D. Winter S. E. Tükel C. Bäumler A. J. (2008). The Vi-capsule prevents Toll-like receptor 4 recognition of *Salmonella*. Cell. Microbiol. 10, 876–890. 10.1111/j.1462-5822.2007.01090.x 18034866

[B58] Winter S. E. Raffatellu M. Wilson R. P. Rüssmann H. Bäumler A. J. (2008). The *Salmonella enterica* serotype Typhi regulator TviA reduces interleukin-8 production in intestinal epithelial cells by repressing flagellin secretion. Cell. Microbiol. 10, 247–261. 10.1111/j.1462-5822.2007.01037.x 17725646

[B59] Yue M. Han X. De Masi L. Zhu C. Ma X. Zhang J.. (2015). Allelic variation contributes to bacterial host specificity. Nat. Commun. 6:8754. 10.1038/ncomms9754 26515720 PMC4640099

[B60] Zhang X. L. Tsui I. S. Yip C. M. Fung A. W. Wong D. K. Dai X.. (2000). *Salmonella enterica* serovar typhi uses type IVB pili to enter human intestinal epithelial cells. Infect. Immun. 68, 3067–3073. 10.1128/IAI.68.6.3067-3073.2000 10816445 PMC97533

