# Supplementary material for: Functional Analysis of the Chaperone-Usher Fimbrial Gene Clusters of Salmonella enterica serovar Typhi
Source: Front Cell Infect Microbiol. 2018 Feb 8;8:26. doi: 10.3389/fcimb.2018.00026 (PMC5809473; doi:10.3389/fcimb.2018.00026)
Supplement: Supplementary file 1 [file Table1.PDF]

Table S1. Bacterial strains and plasmids used for this study

Strain or plasmid	Name	Characteristic	Source or reference
		***S.* Typhi**	
DEF1045	WT	ISP1820 wild-type	R. Curtiss III, U. Florida
DEF004	*Δstg*	WT Δ*stgABCD*	(Forest *et al.*, 2007)
DEF621	*Δsth*	ISP1820 Δ*sthABCDE*	This study
DEF563	*Δbcf*	ISP1820 Δ*bcfABCDEFG*	This study
DEF154	*Δfim*	ISP1820 Δ*fimAICDHFZYXW*	This study
DEF084	*Δsaf*	ISP1820 Δ*safAEBCD*	This study
DEF605	*Δsef*	ISP1820 Δ*sefABCD*	This study
DEF044	*Δsta*	ISP1820 Δ*staABCDEFG::kan*	This study
DEF564	*Δstb*	ISP1820 Δstb*ABCDE*	(Sabbagh *et al*., 2012)
DEF602	*Δstc*	ISP1820 Δ*stcABCD*	(Sabbagh *et al*., 2012)
DEF600	*Δstd*	ISP1820 Δ*stdABC*	This study
DEF603	*Δste*	ISP1820 Δ*steABCDEF*	This study
DEF441	*Δtcf*	ISP1820 Δ*tcfABCD*	(Leclerc *et al.*, 2016)
DEF664	Afimbrial ISP1820	ISP1820 Δ*bcfABCDEFG* Δ*csgCABDEFG* Δ*fimAICDHFZYXW* Δ*pilLMNOPQRSTUVK* Δ*safAEBCD* Δ*sefABCD* Δ*staGFEDCBA::kan* Δ*stbEDCBA* Δ*stcDCBA* Δ*stdCBA* Δ*steABCDEF* Δ*stgABCD* Δ*sthEDCBA* Δ*tcfABCD*	This study
DEF1172	Afimbrial ISP1820 Kan^S	ISP1820 Δ*bcfABCDEFG* Δ*csgCABDEFG* Δ*fimAICDHFZYXW* Δ*pilLMNOPQRSTUVK* Δ*safAEBCD* Δ*sefABCD* Δ*staABCDEFG* Δ*stbABCDE* Δ*stcABCD* Δ*stdABC* Δ*steABCDEF* Δ*stgABCD* Δ*sthABCDE* Δ*tcfABCD*	This study
DEF1153	pRS	WT (pRS415)	This study
DEF1125	pRSstg	WT (pSIF469)	This study
DEF1124	pRSsth	WT (pSIF470)	This study
DEF1130	pRSbcf	WT (pSIF471)	This study
DEF1095	pRSfim	WT (pSIF474)	This study
DEF1090	pRSsaf	WT (pSIF472)	This study
DEF1128	pRSsef	WT (pSIF473)	This study
DEF1082	pRSsta	WT (pSIF464)	This study
DEF1127	pRSstb	WT (pSIF465)	This study
DEF1126	pRSstc	WT (pSIF466)	This study
DEF992	pRSstd	WT (pSIF467)	This study
DEF1131	pRSste	WT (pSIF468)	This study
DEF1132	pRStcf	WT (pSIF219)	This study
DEF1019	pMMB	Afimbrial ISP1820 (pMMB207c)	This study
DEF1106	pMMBstg	Afimbrial ISP1820 (pSIF397)	This study
DEF1133	pMMBsth	Afimbrial ISP1820 (pSIF416)	This study
DEF1113	pMMBbcf	Afimbrial ISP1820 (pSIF395)	This study

DEF1151	pMMBfim	Afimbrial ISP1820 Kan^S (pSIF429)	This study
DEF1114	pMMBsaf	Afimbrial ISP1820 (pSIF412)	This study
DEF1155	pMMBsef	Afimbrial ISP1820 (pSIF432)	This study
DEF1111	pMMBsta	Afimbrial ISP1820 Kan^S (pSIF413)	This study
DEF1107	pMMBstb	Afimbrial ISP1820 (pSIF414)	This study
DEF1112	pMMBstc	Afimbrial ISP1820 (pSIF424)	This study
DEF1207	pMMBstd	Afimbrial ISP1820 Kan^S (pSIF427)	This study
DEF1108	pMMBste	Afimbrial ISP1820 (pSIF396)	This study
DEF1020	pMMBtcf	Afimbrial ISP1820 (pSIF420)	This study
DEF1192	pWSK	Afimbrial ISP1820 Kan^S (pWSK29)	This study
DEF1185	pWSKstg	Afimbrial ISP1820 Kan^S (pSIF026)	This study
DEF1223	pWSKsth	Afimbrial ISP1820 Kan^S (pSIF450)	This study
DEF1228	pWSKbcf	Afimbrial ISP1820 Kan^S (pSIF458)	This study
DEF1224	pWSKfim	Afimbrial ISP1820 Kan^S (pSIF451)	This study
DEF1191	pWSKsaf	Afimbrial ISP1820 Kan^S (pSIF036)	This study
DEF1190	pWSKsef	Afimbrial ISP1820 Kan^S (pSIF444)	This study
DEF1184	pWSKsta	Afimbrial ISP1820 Kan^S (pSIF046)	This study
DEF1187	pWSKstb	Afimbrial ISP1820 Kan^S (pSIF232)	This study
DEF1221	pWSKstc	Afimbrial ISP1820 Kan^S (pSIF445)	This study
DEF1227	pWSKstd	Afimbrial ISP1820 Kan^S (pSIF457)	This study
DEF1222	pWSKste	Afimbrial ISP1820 Kan^S (pSIF446)	This study
DEF1188	pWSKtcf	Afimbrial ISP1820 Kan^S (pSIF119)	This study
DEF1235	WT/pWSK	WT (pWSK29)	This study
DEF1236	WT/pWSKfim	WT (pSIF451)	This study
DEF1232	Δfim/pWSKfim	*Δfim* (pSIF451)	This study
		***E.* coli**	
DEF1162	MGN-617	SM10 λpir asd thi thr leu tonA lacY supE recA RP4 2-Tc : :Mu[λpir] *asdA4*	(Kaniga *et al*., 1998)
		**Plasmids**	
pMEG-375		*sacRB mobRP4 oriR6K*, Cm^r Ap^r	R. Curtiss III, U. Florida
pSIF004		pMEG-375 with flanking region of *stg* operon used for *stg* operon deletion	(Forest *et al.*, 2007)
pSIF210		pMEG-375 with flanking region of *sth* operon used for *sth* operon deletion	This study
pSIF175		pMEG-375 with flanking region of *bcf* operon used for *bcf* operon deletion	(Sabbagh et *al.*, 2012)
pSIF064		pMEG-375 with flanking region of *fim* operon used for *fim* operon deletion	This study
pSIF034		pMEG-375 with flanking region of *saf* operon used for *saf* operon deletion	This study
pSIF206		pMEG-375 with flanking region of *sef* operon used for *sef* operon deletion	This study

pSIF046	pMEG-375 with flanking region of *sta* operon used for *sta* operon deletion	This study
pSIF176	pMEG-375 with flanking region of *stb* operon used for *stb* operon deletion	(Sabbagh et *al.*, 2012)
pSIF198	pMEG-375 with flanking region of *stc* operon used for *stc* operon deletion	(Sabbagh et *al.*, 2012)
pSIF202	pMEG-375 with flanking region of *std* operon used for *std* operon deletion	This study
pSIF203	pMEG-375 with flanking region of *ste* operon used for *ste* operon deletion	This study
pSIF098	pMEG-375 with flanking region of *tcf* operon used for *tcf* operon deletion	(Leclerc *et al.*, 2016)
pRS415	Multicopy vector with a promotorless, *lacZ* reporter gene, Ap^r	(Simons *et al.*, 1987)
pSIF469	pRS415 carrying the promoter region of *stgA*	This study
pSIF470	pRS415 carrying the promoter region of *sthA*	This study
pSIF471	pRS415 carrying the promoter region of *bcfA*	This study
pSIF474	pRS415 carrying the promoter region of *fimA*	This study
pSIF472	pRS415 carrying the promoter region of *safA*	This study
pSIF473	pRS415 carrying the promoter region of *sefA*	This study
pSIF464	pRS415 carrying the promoter region of *staA*	This study
pSIF465	pRS415 carrying the promoter region of *stbA*	This study
pSIF466	pRS415 carrying the promoter region of *stcA*	This study
pSIF467	pRS415 carrying the promoter region of *stdA*	This study
pSIF468	pRS415 carrying the promoter region of *steA*	This study
pSIF219	pRS415 carrying the promoter region of *tcfA*	(Leclerc et al., 2016)
pMMB207c	Wide host range vector, IPTG-inducible	(Morales *et al.*, 1991)
pSIF397	pMMB207-*stgABCD*	This study
pSIF416	pMMB207-*sthABCDE*	This study
pSIF395	pMMB207-*bcfABCDEFG*	This study
pSIF429	pMMB207-*fimAICDHFZYXW*	This study
pSIF412	pMMB207-*safAEBCD*	This study
pSIF432	pMMB207-*sefABCD*	This study
pSIF413	pMMB207-*staABCDEFG*	This study
pSIF414	pMMB207-*stbABCDE*	This study
pSIF424	pMMB207-*stcABCD*	This study
pSIF427	pMMB207-*stdABCD*	This study
pSIF396	pMMB207-*steABCDEF*	This study
pSIF420	pMMB207-*tcfABCD*	(Leclerc et al., 2016)
pWSK29	Low copy number cloning vector, Ap^r	(Wang *et al.*, 1991)
pSIF026	pWSK29 carrying a 5.4 kb fragment of *stgABCD*	(Forest *et al.*, 2007)
pSIF450	pWSK29 carrying a 6 kb fragment of *sthABCDE*	This study

pSIF458	pWSK29 carrying a 7.4 kb fragment of *bcfABCDEFG*	This study
pSIF451	pWSK29 carrying a 9.7 kb fragment of *fimAICDHFZYXW*	This study
pSIF036	pWSK29 carrying a 4.6 kb fragment of *safAEBCD*	This study
pSIF444	pWSK29 carrying a 5 kb fragment of *sefABCD*	This study
pSIF046	pWSK29 carrying a 8 kb fragment of *staABCDEFG*	This study
pSIF232	pWSK29 carrying a 7.1 kb fragment of *stbABCDE*	This study
pSIF445	pWSK29 carrying a 5.2 kb fragment of *stcABCD*	This study
pSIF457	pWSK29 carrying a 5.8 kb fragment of *stdABCD*	This study
pSIF446	pWSK29 carrying a 6.7 kb fragment of *steABCDEF*	This study
pSIF119	pWSK29 carrying a 5.8 kb fragment of *tcfABCD*	(Leclerc et al., 2016)
