# Supplementary material for: Functional Analysis of the Chaperone-Usher Fimbrial Gene Clusters of Salmonella enterica serovar Typhi
Source: Front Cell Infect Microbiol. 2018 Feb 8;8:26. doi: 10.3389/fcimb.2018.00026 (PMC5809473; doi:10.3389/fcimb.2018.00026)
Supplement: Supplementary file 2 [file Table2.PDF]

Table S2. Primers used in this study

**Primers**	**Sequence (5'-3')***
STGA-F	CGGGATCCGAGATGAGAATAACGGAATA
STGA-R	AACTGCAGCCAGCAAATGCCGTTTTGTT
STGD-F	*AA*CTGCAGGCCGCAGAGCTGTGAAATTG
STGD-R	GCTCTAGACATTGATATGACTTATTTTG
SthA F1	CGGGATCCCGTAGAATAGCCGCCTGCTT
SthA R2	*CCGTCAGTT*TGTGGATGCTACGGCAGTTA
SthE F3	*GCATCCACA*AACTGACGGCATCACATTTTC
SthE R4	GCTCTAGAGCCATCTGGACTGGTATTCG
Bcf F1	CGGGATCCACTCACGACGTTGAGTAGCT
Bcf R2	*ACGTCATTC*TGACGGTTGTAGTATCCGCT
Bcf F3	*CAACCGTCA*GAATGACGTGGGAACCTTAG
Bcf R4	AAGGAAAAAAGCGGCCGCTGCTACGCGGTTCAGTCATA
FimAF	CGGGATCCGATATCGAAACCGGGTGTGT
FimAR-over	*ACCTACAGG*GCATATTGGTGCCTTC
FimWR-over	*ATACGCTGC*CCTGTAGGTATCGTTACT
Fim WF	GCTCTAGACTCAGCACGCATAAAGTG
SafA-F	CGGGATCCGACTGGCATTCTATTCCACCCTG
SafA-R	*GTGGACCTG*CAGTTATCATGCTCAACACGCTTG
SafD-F	*TGATAACTG*CACCTGGACGCTGGATTTTAAG
SafD-R	GCTCTAGACCCGCTCACAAAAGAGTGAATC
SefA F1	CGGGATCCGCATCCGCACAGATAAATTG
SefA R2	*GTCTTCTCC*CTGCCTGAACCTCTGCTTTG
SefD F3	*TTCAGGCAG*GGAGAAGACTGGCAACCAGA
SefD R4	GCTCTAGAATGCCGTAGGAATGTCAAGC
StaAF	AACTGCAGGCGTGAAGAGGAAACTACTT
StaAR	GCTCTAGAAAATAATCATGGAACCAGTC
StaGF	AACTGCAGTTATCCAAAGCAGATTATGG
StaGR	CGGGATCCGCGTTAGCCTTTTCTGGGCA
Stb F1	CGGGATCCTGCTGAATTCTGGCCTGTCT

Stb R2	*GTTATTGCC*CGCCGAAAACAGCACTTGAT
Stb F3	*TTTTCGGCG*GGCAATAACACGACGGGTTT
Stb R4	AAGGAAAAAAGCGGCCGCCAGGAGGGTATAGCTCACAT
STY2378-81 F1 (*stc*)	GCTCTAGATGTTGACT GCCTTCACTA CC
STY2378-81 R2 (*stc*)	*GCTGAAATT*AAGCGACTGCGCTGATCTAT
STY2378-81 F3 (*stc*)	*CAGTCGCTT*AATTTCAGCGGTGTTCGTAC
STY2378-81 R4 (*stc*)	AAGGAAAAAAGCGGCCGCGCGATAACTTCCTGTCTATG
StdA F1	CGGGATCCCGATGGAAAGTTCAGGTGCT
StdA R2	*TTAAGGGCA*CCGCCATGGCAAGTATTATT
StdC F3	*CCATGGCGG*TGCCCTTAAAGGCTGTTCTG
StdC R4	GCTCTAGAATACCTGGCTCAACCGCATA
SteA F1	CGGGATCCCTATGCCGCATATCCCTTGA
SteA R2	*CTCTGCCAA*CCGGAGACAATTCCCATAAC
SteD F3	*TGTCTCCGG*TTGGCAGAGGGAAATACCAT
SteD R4	GCTCTAGACCAGAGCATCAATGCCTTT
TcfAF prom	GCTCTAGACATGATGATCAGTCTATTTGTGGC
TcfAR over	*TGTCAGGGT*AATTTCTGCCGCCATGGGATA
TcfDF over	*GCAGAAATT*ACCCTGACAACACAACCCTT
TcfDR	AAGGAAAAAAGCGGCCGCAGCAGAACCTCACGCATTGA
Stg_Prom_F_EcoRI	GCGAATTCCGGGAGATGAGAATAACGGA
Stg_Prom_R_BamHI	GTGGATCCAGTAGAAGACAGAACCAGAGCG
Sth_Prom_F_EcoRI	GCGAATTCAAATCCAGTCATCTACCGTACTTC
Sth_Prom_R_BamHI	GCGGATCCGTGGATGCTACGGCAGTTAACA
Bcf_Prom_F_Short_EcoRI	GTGGATTCAACTCACGACGTTGAGTAGCTG
Bcf_Prom_R_BamHI	GCGGATCCCAACATTCCGCCAAAGGCAA
Fim_Prom_F_Long_EcoRI	AGGAATTCCTTCAAGTCAAAGGGGATAACGCT
Saf_Prom_F_EcoRI	CAGAATTCTGTTATTACCAGCCAGGGAT
SafA R2	GTCCAGGTGCAGTTATCATGCTCAACACGCTTG
Sef_Prom_F_EcoRI	GCGAATTCCTTAGTCGCATGTCCACTCTTGCT
Sef_Prom_R_BamHI	ACGGATCCTGGGCACTGCCACATGCAATTA
Sta_Prom_F_EcoRI	GCGAATTCCAGCCGCTTAATGCAATTAAACA
Sta_Prom_R_BamHI	ATGGATCCTGCGGCAGCTAAAATCGCTT

Stb_Prom_F_Short_EcoRI	GCGAATTCCATCGGGAGGTTTAACTGATACGG
Stb_Prom_R_BamHI	GCGGATCCGCGAGCCTGTGATCATTGCTAAATA
Stc_Prom_F_EcoRI	GCGAATTCAATTCCGCAGGCCCATATCA
Stc_Prom_R_BamHI	GCGGATCCCCCAGCGCTCATAAATACAGCA
Std_Prom_F_EcoRI	GCGAATTCGTTCTTTGCTGTCGGCATTG
Std_Prom_R_BamHI	AAGGATCCACAGAAGCGCCATACATCATACCG
Ste_Prom_F_2_EcoRI	GCGAATTCACAGGTGTTGAATGCTACCTTTCCC
Ste_Prom_R_BamHI	GCGGATCCGCCGGAGACAATTCCCATAACTAAA
TcfAR prom	AATTTCTGCCGCCATGGGATA
Stg_Operon_F_SacI	GCGAGCTCAGGAAACAGACCATGAAACTGAATTTAATTGC
Sth_Operon_F_BamHI	GCGGATCCAGGAAACAGACCATGTTTAATAAGAAAATTATCA
Bcf_Operon_F_SacI	CGGAGCTCACCAAACAGACCATGAAAAAGCCTGTACTAGCA
Bcf_Operon_R_XbaI	GCTCTAGACGTTCGATAAGATCGGAAAG
Fim_Operon_F2_BamHI	GCGGATCCAGGAAACAGACCATGAAACATAAATTAATGACC
Fim_Operon_R3_XbaI	GCTCTAGATCAGGCACTCCTGAGTCAAT
Saf_Operon_F_EcoRI	GCGAATTCAGGAAACAGACCATGAAAAACATAAAAAAAT
Saf-RC	GCTCTAGATGCTAACCATATTTGCCTTGAG
Sef_Operon_F_EcoRI	AGGAATTCAGGAAACAGACCATGCGTAAATCAGCATC
SefD_R4_SacI	GCTCTAGAATGCCGTAGGAATGTCAAGC
Sta_Operon_F_SacI	CGGAGCTCAGGAAACAGACCATGAAAAAAGCGATTTTAGC
Sta_Operon_R_XbaI	ACTCTAGAATTGCGTTGCGGTTCGTT
Stb_Operon_F_SacI	CGGAGCTCAGGAAACAGACATGTCTATGAAAAAATATTTA
Stb_Operon_R_XbaI	GCTCTAGATGCTGAATTCTGGCCTGTC
Stc_Operon_F_XbaI	GCTCTAGAAGGAAACAGACCATGAAACGTTCACTTATT
Stc_Operon_R_SalI	CGGTCGACAGATTGTCATCCCGGTCACT
Std_Operon_F4_SacI	CGAGCTCATGCGTAATAAAATAATACTTGCC
Std_Operon_R3_XbaI	GCTCTAGAATTAGTTCCCCGATAACTCAGTCA
Ste_Operon_F_SacI	GCGAGCTCAGGAAACAGACCATGAAGTCATCTCATTTTTG
Tcf_Operon_F_SacI	CGCGAGCTCAAGAAACAGACCATGAATTTTAAAGATACTCTTCC
Tcf_Operon_R_XbaI	GCTCTAGAAAAAACCATATAAGAAAGATATCAA
Sth_Prom_F_BamHI	CGGGATCCAAATCCAGTCATCTACCGTACTTC
Fim_Prom_F_Short_BamHI	CGGGATCCGGCGGCATAATGCGACATTT

Saf-FC	CCCTCGAGGGAAGATAAGTTTCCCACACCC
Stc_Prom_F_XbaI	GCTCTAGAAATTCCGCAGGCCCATATCA
Std_Prom_F_SacI	CGAGCTCGTTCTTTGCTGTCGGCATTG
Ste_Prom_F_2_BamHI	CGGGATCCACAGGTGTTGAATGCTACCTTTCCC

*Restriction enzyme sites are underlined. Letter in italics represent overlapping sequences.
