# Supplementary figures and images for: Functional Analysis of the Chaperone-Usher Fimbrial Gene Clusters of Salmonella enterica serovar Typhi
Source: Front Cell Infect Microbiol. 2018 Feb 8;8:26. doi: 10.3389/fcimb.2018.00026 (PMC5809473; doi:10.3389/fcimb.2018.00026)

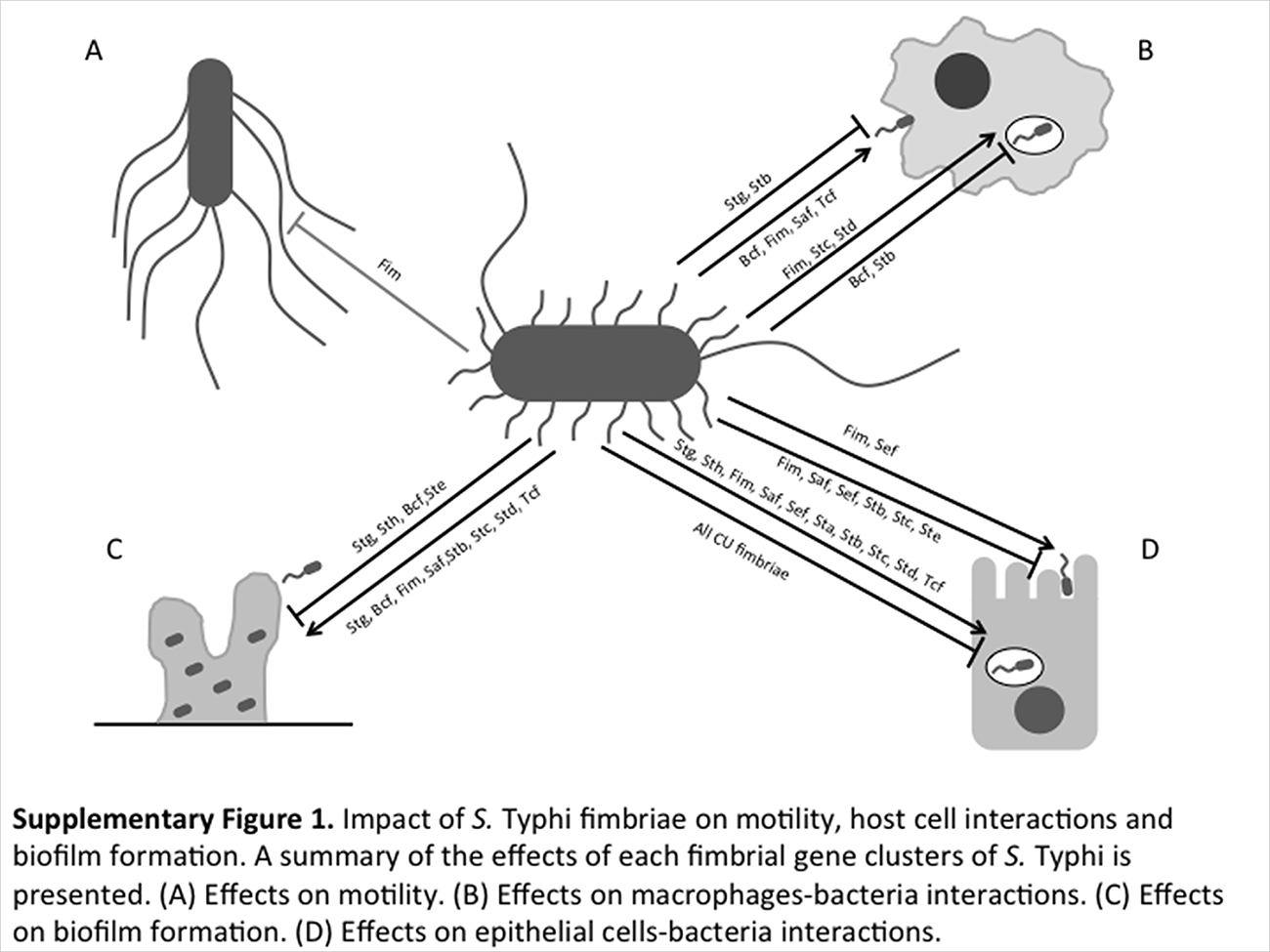

Supplement: Supplementary file 3 [file Image1.TIFF]
